# Development of an atomic cluster expansion potential for iron and its oxides

**DOI:** 10.1038/s41524-025-01574-w

**Published:** 2025-03-26

**Authors:** Baptiste Bienvenu, Mira Todorova, Jörg Neugebauer, Dierk Raabe, Matous Mrovec, Yury Lysogorskiy, Ralf Drautz

**Affiliations:** 1 Max Planck Institute for Sustainable Materials, Max-Planck-Straße 1, 40237 Düsseldorf, Germany; 2 Interdisciplinary Centre for Advanced Materials Simulations, Ruhr Universität Bochum, 44780 Bochum, Germany

**Keywords:** Atomistic models, Metals and alloys

## Abstract

The combined structural and electronic complexity of iron oxides poses many challenges to atomistic modeling. To leverage limitations in terms of the accessible length and time scales, one requires a physically justified interatomic potential which is accurate to correctly account for the complexity of iron-oxygen systems. Such a potential is not yet available in the literature. In this work, we propose a machine-learning potential based on the Atomic Cluster Expansion for modeling the iron-oxygen system, which explicitly accounts for magnetism. We test the potential on a wide range of properties of iron and its oxides, and demonstrate its ability to describe the thermodynamics of systems spanning the whole range of oxygen content and including magnetic degrees of freedom.

## Introduction

Iron is one of the most abundant elements on Earth and is the primary constituent in steels and other metallic alloys. In both its natural and processed appearances, it is often present in the form of oxides, which develop easily under ambient conditions. A thorough understanding of the properties of iron oxides down to the atomic and electronic levels is therefore crucial for optimizing the production of pure iron from its ores as well as for minimizing the impact of environmental degradation due to oxidation.

The Fe-O binary system, although it contains only a few stable compounds, reveals a wide range of structural and electronic variety and complexity^1^. Iron oxides are mainly found in nature in the form of three stable minerals, namely, wüstite (FeO), magnetite (Fe_3_O_4_) and hematite (Fe_2_O_3_), by increasing oxygen concentration.

Most atomistic studies of the Fe-O system were carried out using Density Functional Theory (DFT) calculations. However, a faithful description of the respective compounds requires the use of different exchange and correlation (xc-) functionals depending on the oxygen content in the structure of interest. Indeed, standard semi-local DFT functionals such as LDA or various GGAs predict not only Fe but also its oxides to be metallic, which is in disagreement with experimental observations. This discrepancy has motivated further investigations of the oxides with more sophisticated methodologies, such as DFT + *U* or hybrid functionals^2^.

First-principles calculations face limitations regarding the size of simulated systems and the time scales of simulations. Therefore, they are impractical for exhaustive studies of extended defects, such as grain boundaries, interfaces, dislocations, or kinetic processes, such as diffusion, oxidation, and phase transformations. Atomistic modeling of such complex phenomena necessitates an interatomic potential that is both accurate and efficient. However, the development of robust and transferable potentials that would be able to capture the Fe-O system in its entirety over a broad range of temperatures, stoichiometries, stresses, chemical potentials, etc., presents a major challenge.

Most of the available interatomic potentials for the Fe-O system are based on the ReaxFF formalism^3^ and exhibit severe discrepancies both with DFT and experiments in terms of structural and transport properties^4^. Most importantly, the parameterization of Aryanpoor et al.^5^ and subsequent models predict the three iron oxides to be dynamically unstable. An analytical bond order potential (ABOP) was developed recently^6^ to investigate oxygen defects in BCC Fe and the stoichiometric wüstite phase. However, due to its limited transferability, it yields non-physical results for phases with higher oxygen content, with Fe_3_O_4_ and Fe_2_O_3_ melting already at room temperature^6^.

In the present work, we develop a machine-learned interatomic potential (MLIP) based on the atomic cluster expansion (ACE)^7^ that explicitly accounts for magnetism, which is crucial for a physically correct description of the Fe-O system. The potential is fitted to an extensive DFT database encompassing pure Fe as well as a large variety of oxygen-containing phases. We validate the ACE parametrization for a wide range of properties of both pristine and defective phases spanning the whole range of oxygen concentrations. We also demonstrate the ability of the model to provide an explicit description of magnetic degrees of freedom and behavior at finite temperatures.

## Results

### DFT reference data

**Fig. 1:**
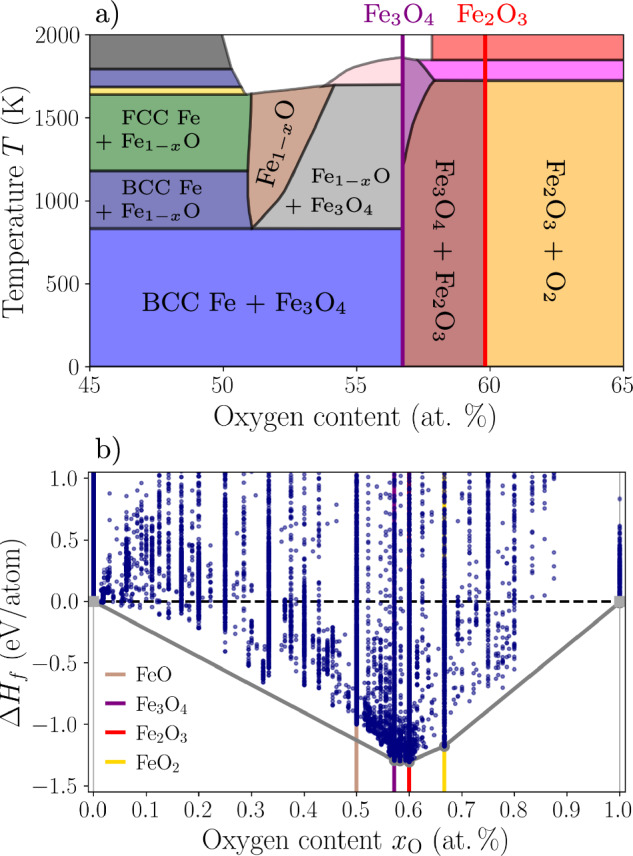
Experimental phase diagram and DFT reference dataset. **a** Experimental Fe-O phase diagram adapted from ref. ^8^ showing the stable compounds. **b** DFT convex Hull (i.e., formation enthalpy Δ*H*_*f*_ as a function of the oxygen atomic concentration *x*_O_) computed in this work.

The intended applicability range of the ACE potential spans the entire experimental Fe-O binary phase diagram^8^ presented in Fig. 1a, from pure Fe phases to highly oxidized environments encompassing the stability ranges of the three iron oxides.

At low pressure and temperature, pure Fe crystallizes in a body-centered cubic (BCC) phase with a ferromagnetic (FM) order. This structure is stable up to approximately 1200 K, where the BCC FM phase destabilizes in favor of a disordered paramagnetic (PM) face-centered cubic (FCC) phase. At higher pressures, pure Fe takes a hexagonal close-packed (HCP) structure, which remains stable up to extremely high pressures^9^.

In the binary Fe-O system, up to its solubility limit, O atoms occupy interstitial sites in the host Fe lattice. The first ordered oxide above this limit, wüstite Fe_1−*x*_O, appears in the phase diagram at approximately 50% of oxygen and is characterized by a degree of Fe deficiency *x* varying between 17 and 5%^10^. Wüstite has a rock-salt structure (NaCl, ${\rm{Fm}}\overline{3}{\rm{m}}$), with two displaced FCC sublattices of Fe and O and an antiferromagnetic (AF) order at low temperature. At 57% oxygen, the most stable oxide becomes magnetite, Fe_3_O_4_, which crystallizes under ambient conditions in an inverse spinel structure (${\rm{Fd}}\overline{3}{\rm{m}}$) with a ferrimagnetic order. In Fe_3_O_4_, the O atoms are arranged in an FCC lattice, with the Fe atoms occupying both the octahedral and tetrahedral interstitial sites while having opposite spin directions on the two sites. Above 60% oxygen, Fe_2_O_3_ is the primary stable oxide, with a corundum structure (${\rm{R}}\overline{3}{\rm{c}}$), an AF magnetic order, and an HCP underlying O sublattice^1^.

In terms of electronic properties, under ambient temperature and pressure, Fe_1−*x*_O and Fe_2_O_3_ are insulators, while Fe_3_O_4_ is a half-metal^1^. The distinct electronic structures add a further degree of complexity to the structural and magnetic complexity highlighted above.

Carrying out atomistic simulations of iron oxides at the DFT level is challenging due to a high degree of electronic correlations of the *d*-orbitals on Fe atoms. A proper description of the electronic properties thus needs to rely on a formalism that is able to incorporate electronic correlations to some extent, for instance, via the widely used framework of DFT + *U*^11^. However, a better agreement with experiments in terms of electronic properties^2^ is counteracted by a worse description of structural and thermodynamic properties; for instance, predicting an almost zero diffusion barrier for oxygen interstitials in BCC Fe^12^ or incorrect ordering of the BCC and FCC Fe phases (see Supplementary Materials). This is partly due to the fact that the *U* correction is an empirical parameter, which is usually tuned to reproduce a given property of interest (e.g., the electronic band gap), and is thus material-dependent^2^. A common compromise to obtain properties of both pure Fe and its oxides within DFT is to use DFT + *U* for oxides and a standard functional, such as GGA-PBE^13^, for pure Fe. For instance, an empirical mixing scheme was implemented in the Materials Project^14^ to obtain formation enthalpies comparable to experimental results^15^.

However, in the context of MLIP fitting, a key feature of the underlying DFT reference data is its coherency in terms of DFT parameters. In the case of the Fe-O system, this is primarily the xc-functional. Thus, to maintain a consistent description of Fe atoms across the whole range of oxygen concentrations, a single DFT functional must be chosen. Since GGA-PBE gives a good agreement with experiments in terms of structural properties for all three oxides (lattice constants and elastic properties, see Table 1) as well as a reliable description of pure Fe phases, we chose to use this xc-functional to compute the whole DFT dataset.

**Table 1:** Bulk properties of Fe and its oxides predicted by ACE and compared to DFT calculations (with and without *U* correction) and experiments (extrapolated to 0 K): lattice constants (*a*_0_ for cubic lattices, *a* and *c* for hexagonal Fe_2_O_3_), bulk modulus *B*_0_, and 0 K formation enthalpy Δ*H*_*f*_

	DFT			
	ACE	GGA-PBE	+*U*_Fe_ = 4 eV	Expt.
Iron, Fe (BCC, FM)				
a_0_ (Å)	2.84	2.83	2.95	2.86^55^
B_0_ (GPa)	165	188	123	164^9^
Stoichiometric wüstite, FeO (distorted NaCl, AF)				
a_0_ (Å)	4.29	4.26	4.31	4.33^56^
B_0_ (GPa)	181	195	164	175^56^
Δ*H*_*f*_ (eV/atom)	−0.95	−0.91	−1.43	−1.41^23^
Magnetite, Fe_3_O_4_ (inverse spinel, FeM)				
a_0_ (Å)	8.40	8.40	8.47	8.40^57^
B_0_ (GPa)	165	172	191	183^57^
Δ*H*_*f*_ (eV/atom)	−1.29	−1.29	−1.75	−1.66^23^
Hematite, Fe_2_O_3_ (corundum, AF)				
* a* (Å)	4.98	5.02	5.07	5.04^58^
* c* (Å)	14.06	13.90	13.90	13.75^58^
* B*_0_ (GPa)	156	172	191	225^58^
Δ*H*_*f*_ (eV/atom)	−1.30	−1.30	−1.81	−1.71^23^

For each structure, the lowest energy crystal structure and magnetic order are indicated (FM for ferromagnetic, AF for antiferromagnetic, and FeM for ferrimagnetic). The DFT + *U* values for Δ*H*_*f*_ are obtained without applying the correction schemes of refs. ^15,59^. Experimental data are taken from various references, indicated for each property and material.

Since Fe and its oxides exhibit ordered magnetic states from 0 K up to relatively high temperatures, magnetism must be accounted for in all DFT calculations. The Curie temperature of Fe is around 1000 K, where the BCC FM order transitions to a disordered PM state. For both Fe_3_O_4_ and Fe_2_O_3_, the Néel temperature marking the transition to the PM state is about 950 K^16,17^. For wüstite, the Néel temperature is around 200 K^1^, depending on the degree of Fe depletion *x*^18^. Thus, for applications such as catalysis^19^ and redox reactions below 1000 K, the explicit consideration of magnetism for both iron and its oxides is essential. For the present ACE potential, the description of magnetism is based on collinear spin-polarized DFT calculations. More details about the DFT parameters are given in the “Methods” section.

The phases occurring in the experimental Fe-O phase diagram presented in Fig. 1a, namely, BCC and FCC Fe, Fe_1−*x*_O, Fe_3_O_4_, and Fe_2_O_3_, constitute the foundation for the DFT reference data. Pure oxygen is included through sampling of its gas and liquid phases only and to a much lesser extent compared to structures containing up to 80% oxygen. It should be noted that the description of chemical bonding in molecular oxygen by the GGA-PBE xc-functional is also rather poor^20^.

All prototype structures were distorted by applying random strains to the unit cells to sample their elastic properties and deformation behavior. Additionally, atoms were randomly displaced to account for thermal vibrations. Apart from these distorted structures, the most relevant defects were generated, including vacancies, interstitials (both Fe and O), clusters composed of the two types of point defects, and surfaces of various low-index orientations. Furthermore, several grain boundaries and dislocation configurations were considered for pure Fe in its ground-state BCC structure.

Finally, to sample a wider part of the configuration space, both in terms of atomic environments and oxygen concentrations, we also included randomly generated structures in the training data. These structures were constructed such that they respected constraints on their point-group symmetry, number of atoms, and oxygen concentration^21^. These “random” structures are of great importance for the parametrization of the present ACE potential since they inform the model about out-of-equilibrium and highly distorted configurations that are relevant for the dynamics of complex defects, phase transformations, and liquid phases. It has been shown that including such structures in the reference database drastically increases the reliability and transferability of MLIPs^22^. Since the aim of the present ACE potential is to describe structures that are, for the most part, not known a priori and span the whole range of oxygen concentration, it is important that the model can interpolate between data points for a wide variety of atomic environments. These structures make up the majority of the overall database.

To capture the magnetic behavior, we also sampled the magnetic degrees of freedom by considering different collinear magnetic orders for all prototype materials (i.e., Fe and its oxides) as well as for the random structures. The final DFT reference database is presented in Fig. 1b, showing the formation enthalpy Δ*H*_*f*_ of all structures as a function of the oxygen concentration *x*_O_. It can be seen that stoichiometric wüstite (FeO) lies above the DFT convex hull, and is thus predicted to be thermodynamically unstable at 0 K in agreement with experiments, unlike in DFT + *U* calculations^2^. For the non-stoichiometric wüstite, the comparison with experiments is difficult, since the cation defect structure in Fe_1−*x*_O is extremely complicated and still under debate^10^. The lowest formation enthalpy is found for Fe_2_O_3_, in agreement with experiments^23^. We also report the stability of FeO_2_, a compound observed experimentally at high temperature and pressure^24^. Finally, we note that temperature-composition phase transitions present in the experimental phase diagram of Fig. 1a are not discussed in this study.

### Potential parameterization

The Atomic Cluster Expansion^7^ provides a physically justified and complete basis set for the description of atomic environments. Potentials based on ACE have proven to be robust and versatile to capture different types of interatomic interactions, including covalent^25^, metallic^26,27^ and mixed^28,29^ chemical bonding. ACE is also able to incorporate additional vectorial degrees of freedom, such as magnetic moments. This was demonstrated recently for Fe where a non-collinear magnetic ACE model^30^ showed an excellent description of magnetic excitations, allowing to accurately predict the transition temperatures from BCC to FCC driven by magnetic disorder. For a detailed description of the ACE formalism, the reader is referred to refs. ^7,26,31^.

As discussed above, most materials of interest in the Fe-O phase diagram show ordered magnetic states up to temperatures of around 1000 K^1^. An explicit account of magnetism in the model therefore appears necessary to reliably describe the system at various temperatures.

In this work, we incorporated the magnetic degrees of freedom in the ACE model in a rudimentary but adequate way by using an Ising-like description. This was done by considering three different types of Fe atoms defined according to the sign of their magnetic moments: spin up (Fe_*↑*_), spin down (Fe_*↓*_), and non-magnetic (Fe_NM_). A threshold absolute value of 0.1 *μ*_B_ was set to distinguish between these three types of Fe atoms. Such a simple model for magnetism does not provide an accurate representation of complex magnetic excitations at finite temperatures, which are mostly non-collinear. However, it allows for an explicit account of magnetism at a computational cost and complexity suitable for large-scale simulations of magnetic systems, where both atomic positions and magnetic moments can evolve. The more accurate and thorough account of magnetic degrees of freedom^30^ would require the use of constrained non-collinear spin-polarized DFT calculations and a thorough sampling of non-equilibrium magnetic degrees of freedom. As we aim at a general-purpose binary potential describing a wide variety of atomic environments, the number of configurations that would have to be computed is currently prohibitive.

The magnetic structures are included twice in the training set, considering in each case opposite spin configurations (for instance, FM order with all spins up and all spins down) to enforce the inversion symmetry during the fitting procedure detailed in the next section. Since the data augmentation only is not sufficient to strictly guarantee the spin inversion symmetry, the interaction parameters between magnetic Fe species were additionally symmetrized after the training. All presented results were obtained using this potential. The complete training dataset, including the inverted magnetic structures, contained approximately 40,000 entries.

The cutoff distance common to all interactions was set to 7.0 Å, which we find is necessary to capture the range of interactions between Fe and O atoms in various bonding environments. The present model contains 1000 functions per element, which, including all three types of iron atoms (Fe_*↑*_, Fe_*↓*_, and Fe_NM_) and oxygen, results in a total of 4000 functions and 10352 parameters. The optimization of the parameters was done using the PACEMAKER code^26,31^.

The overall accuracy of the potential across the entire database (in mean absolute errors) is 20 meV/atom and 73 meV/Å in energies and force components, respectively. During the fitting procedure, structures lying within 0.1 eV/atom of the Fe-O DFT convex Hull (see Fig. 1b) were given greater weights, resulting in an improved accuracy of 13 meV/atom and 65 meV/Å in energies and force components, respectively, in this region. These seemingly large errors mostly originate from trying to fit complex magnetic excitations with a simple model for magnetism. We will show in the following that however large the magnitude of the reported error metrics seems, it does not reflect the accuracy of the ACE potential in terms of the predicted properties.

### Validation of bulk properties

In this section, we validate the accuracy of the present ACE potential in predicting bulk properties of both Fe and its oxides by comparing the results to both DFT data and experiments. We also compare the results obtained with the ACE potential to the predictions of the two Fe-O interatomic potentials available from the literature, namely, the ABOP potential from Byggmastar et al.^6^ referred to as ABOP2019, and the ReaxFF potential of Aryanpoor et al.^5^ referred to as ReaxFF2010.

A summary of the bulk properties of pure Fe and its three oxides (lattice constants, bulk moduli, and formation enthalpies) is presented in Table 1. A very good agreement between the properties predicted by ACE and both DFT and experimental references is obtained in terms of lattice constants and bulk moduli for all four materials. The main discrepancy between ACE and experiments, which is also reflected in the underlying DFT data used for its fitting, is the underestimation of the 0 K formation enthalpies of the iron oxides. This discrepancy can be partially resolved by using DFT + *U* as discussed above. Hereby, a value of *U*_Fe_ = 4 eV was chosen for comparison since it has been shown to give the most satisfactory agreement with experiments for all three oxides in terms of electronic properties and formation energies^2^. Otherwise, we also note that for structural properties, GGA-PBE, and thus the ACE potential, are in better agreement with experiments than predictions of DFT + *U*.

**Fig. 2:**
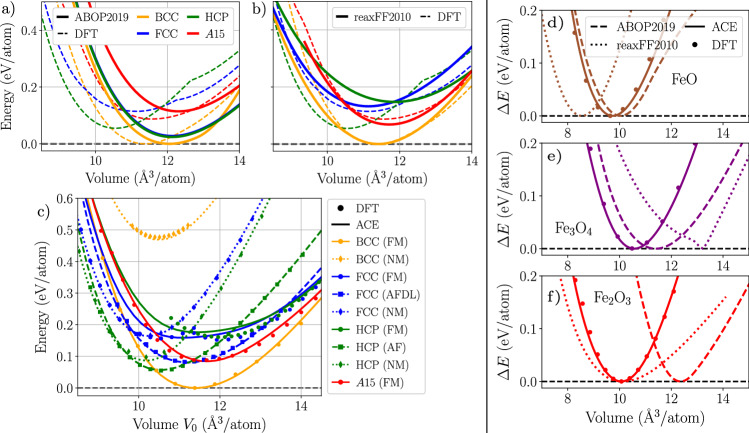
Energy-volume for iron and iron oxides. **a**–**c** Energy-volume curves for different crystal structures for pure Fe, namely BCC, FCC, HCP, and *A*15, comparing DFT (dashed lines) with **a** ABOP2019, **b** ReaxFF2010, and **c** the present Fe-O ACE potential. For the DFT data, only the magnetic ground state is plotted at each volume in (**a**) and (**b**) since the two potentials cannot distinguish between different magnetic orders. This is reflected in the kinks present in the DFT curves for FCC and HCP structures, corresponding to a change in the lowest energy magnetic order. In (**c**), different magnetic orders (AFDL: antiferromagnetic double layer; NM: non-magnetic) are shown when comparing DFT to ACE since the potential captures magnetic degrees of freedom. **d**–**f** Energy-volume curves for the three iron oxides: **d** stoichiometric wüstite FeO, **e** magnetite Fe_3_O_4_, and **f** hematite Fe_2_O_3_ obtained with the ABOP2019 (dashed lines), ReaxFF2010 (dotted lines), ACE (full lines) potentials, and DFT (GGA-PBE, symbols).

We present in Fig. 2 the variation of the energy as a function of the atomic volume for different crystal structures and magnetic orders of Fe and the three FeO, Fe_3_O_4_ and Fe_2_O_3_ oxides. We report a very good agreement between the DFT reference and the predictions of the ACE potential, capturing the hierarchy between both different crystal structures and magnetic orders, as exemplified in the case of pure Fe (see Fig. 2c). This complex intertwining of phases is described by neither ABOP2019 nor ReaxFF2010, as presented in Fig. 2a, b, respectively. These two models are unable not only to differentiate between different magnetic phases but also show large discrepancies in energetical ordering and equilibrium volumes of the Fe phases.

The ACE predictions agree very well with the DFT reference also for the three oxides, showing its applicability to both metallic bonding in pure Fe and mixed bonding in the oxides. We also computed phonon spectra for the stable polymorphs and compared them to those of DFT calculations. These results, shown in Supplementary Figs. 2 and 6, confirm a very satisfactory description of the vibrational properties of the ACE potential. In contrast, ReaxFF2010 and ABOP2019 give satisfactory descriptions for FeO only. For Fe_3_O_4_, ReaxFF2010 shows a non-physical *E*−*V* dependence. ABOP2019 predictions for Fe_3_O_4_ and Fe_2_O_3_ are less reliable since the potential was not trained to reproduce their properties. Apart from discrepancies in bulk moduli, the authors reported these two oxides to melt already at room temperature. Finally, we found that all three oxides are dynamically unstable when computed using ReaxFF2010. For these reasons, further comparisons with ReaxFF2010 and ABOP2019 potentials will be omitted when discussing defect properties in iron and its oxides.

### Point defects and diffusion

**Fig. 3:**
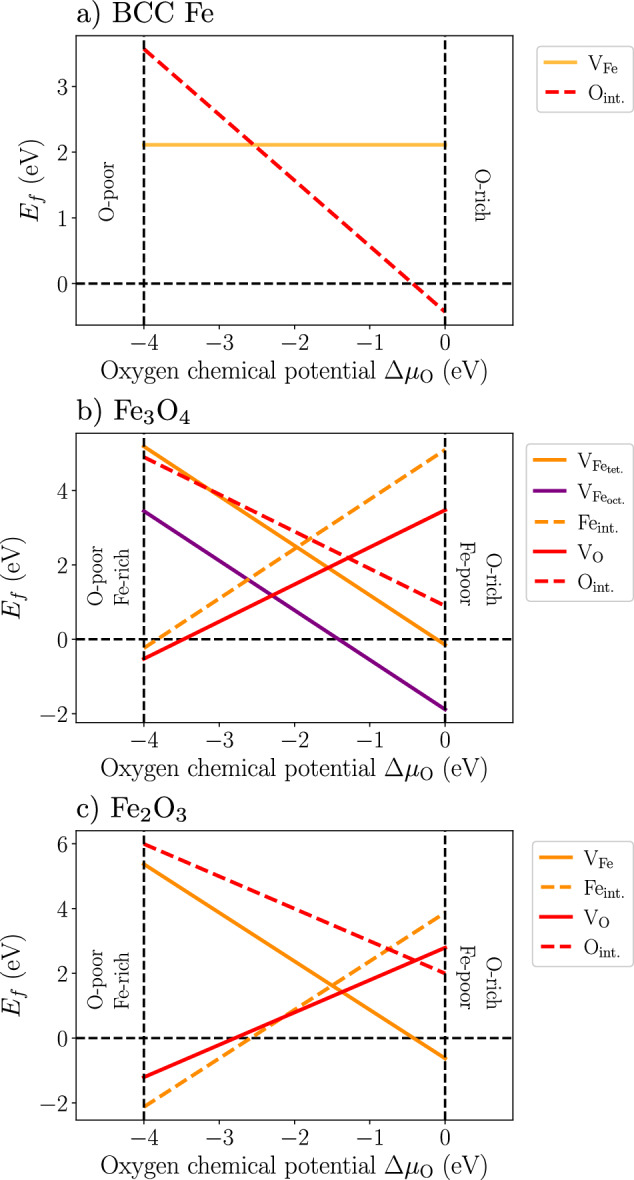
Point defect formation energies in iron and iron oxides. Point defect formation energies *E*_*f*_ as a function of the oxygen chemical potential Δ*μ*_O_ in (**a**) BCC Fe (O octahedral interstitials only), **b** Fe_3_O_4_, and **c** Fe_2_O_3_ predicted by ACE. Δ*μ*_O_ is referenced with respect to O in the O_2_ molecule.

The formation energies of vacancies and interstitials as a function of the oxygen chemical potential Δ*μ*_O_ (referenced to the energy of an O atom in molecular O_2_) predicted by ACE are presented in Fig. 3 for BCC Fe, Fe_3_O_4_, and Fe_2_O_3_. Wüstite, and in particular its stoichiometric form FeO, is not included since it is unstable at 0 K. Details about the evaluation of the formation energies are given in the “Methods” section. It is worth noting that in both DFT and the present ACE potential, the effect of charges is not included. It has been shown recently that charge variations may influence the formation energies of point defects in oxides with respect to the neutral case, particularly for Fe vacancies and interstitials^32^.

**Fig. 4:**
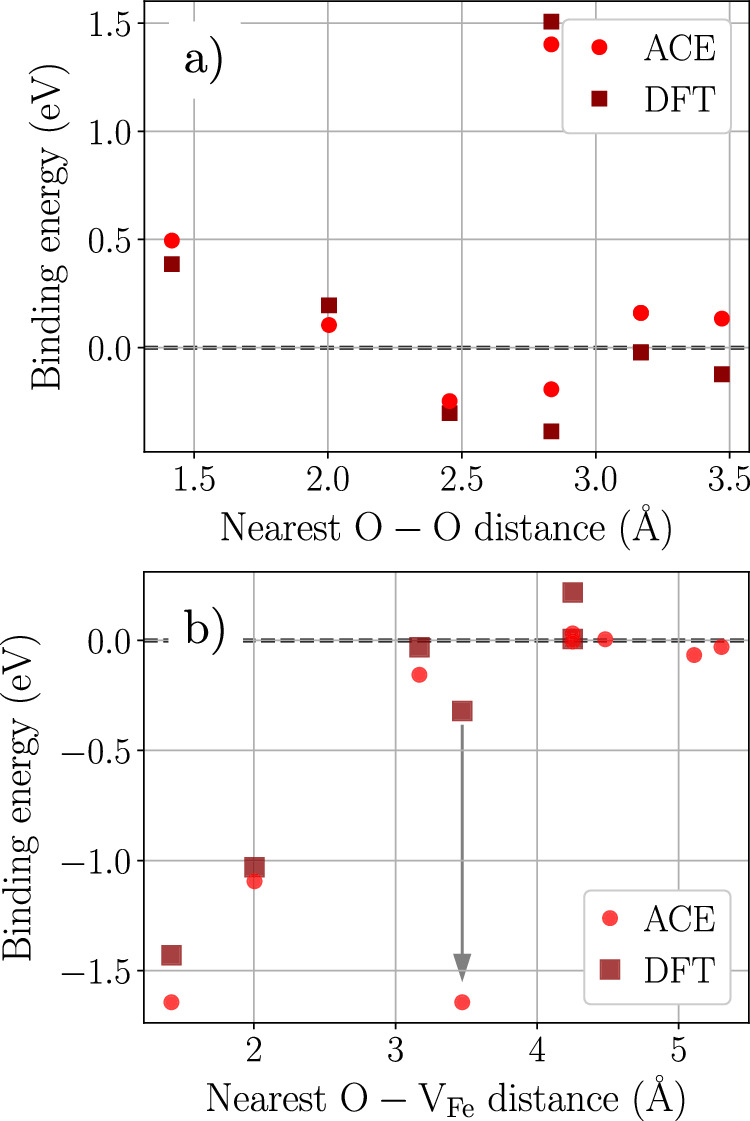
Oxygen/vacancy binding energies in BCC Fe. Binding energies between **a** two octahedral O interstitials, and **b** one O octahedral interstitial and an Fe vacancy as a function of the distance between the two point defects. DFT data in (**b**) are taken from ref. ^34^. The gray arrow at the position of 4th nearest neighbor indicates its relaxation to a 1st nearest neighbor configuration.

The formation energy of a single vacancy in BCC Fe is 2.11 eV according to ACE, showing a close agreement with the DFT reference of 2.17 eV and the experimental value of 2.0 ± 0.2 eV^33^. The formation energy of an oxygen interstitial in BCC Fe, which prefers to occupy the octahedral sites^34^, is also in very good agreement with the reference DFT value and the literature data. We also present in Fig. 4 the binding energy between two O interstitial atoms in BCC Fe, as well as the binding energy between one O interstitial atom and a Fe vacancy. These properties play a key role in the diffusion of both oxygen in iron^12,34^ and in the formation of oxides in the oxygen-rich region^35^.

We report a very satisfactory agreement between ACE and available DFT reference for both O-O and O-vacancy binding energies, reproducing the strongly attractive and short-range interaction between oxygen atoms and vacancies in BCC Fe, as well as the repulsive interaction between neighboring oxygen interstitials. For the 4th nearest neighbor site between an octahedral O and a Fe vacancy (distance of approximately 3.5 Å in Fig. 4b), ACE predicts the configuration to be unstable and relaxes to the 1st nearest neighbor position. The latter was also reported in another DFT study^36^.

Table 2 summarizes the 0 K migration barriers of Fe vacancies and O interstitials for different pathways obtained using ACE together with available DFT data. The migration energy of a single Fe vacancy is 0.69 eV, in good agreement with previous DFT studies^30,37^.

**Table 2:** Height of diffusion barriers (in eV) for migration of O interstitial atoms and Fe vacancy in BCC Fe (FM order): O_oct._ − O_oct._ corresponds to the jump of an O interstitial atom between two adjacent octahedral sites; ${{\rm{O}}}_{{\rm{oct.}}}^{{\rm{V}},1{\rm{NN}}}-{{\rm{O}}}_{{\rm{oct.}}}^{{\rm{V}},1{\rm{NN}}}$ corresponds to the same jump but between two adjacent octahedral sites that are nearest neighbors to a Fe vacancy; $1{{\rm{V}}}_{{\rm{Fe}}}^{1{\rm{NN}}}-1{{\rm{V}}}_{{\rm{Fe}}}^{1{\rm{NN}}}$ corresponds to the migration of a single Fe vacancy; $2{{\rm{V}}}_{{\rm{Fe}}}^{1{\rm{NN}}}-2{{\rm{V}}}_{{\rm{Fe}}}^{1{\rm{NN}}}$ is the collective migration of a di-vacancy, and $3{{\rm{V}}}_{{\rm{Fe}}}^{1{\rm{NN}}}-3{{\rm{V}}}_{{\rm{Fe}}}^{1{\rm{NN}}}$ of a tri-vacancy

	ACE	DFT
O_oct._ − O_oct._	0.42	0.46, 0.56^60^, 0.53^12^, 0.51^36^
${{\rm{O}}}_{{\rm{oct.}}}^{{\rm{V}},1{\rm{NN}}}-{{\rm{O}}}_{{\rm{oct.}}}^{{\rm{V}},1{\rm{NN}}}$	0.56	0.57, 0.59^36^
$1{{\rm{V}}}_{{\rm{Fe}}}^{1{\rm{NN}}}-1{{\rm{V}}}_{{\rm{Fe}}}^{1{\rm{NN}}}$	0.69	0.70, 0.67^30,37^
$2{{\rm{V}}}_{{\rm{Fe}}}^{1{\rm{NN}}}-2{{\rm{V}}}_{{\rm{Fe}}}^{1{\rm{NN}}}$	0.67	0.62^37^
$3{{\rm{V}}}_{{\rm{Fe}}}^{1{\rm{NN}}}-3{{\rm{V}}}_{{\rm{Fe}}}^{1{\rm{NN}}}$	0.21	0.35^37^

The diffusion barrier of 0.42 eV predicted by ACE for the migration of an O interstitial atom between two adjacent octahedral sites (via the unstable tetrahedral site), compares very well with the DFT reference value of 0.46 eV computed in this work as well as with the values reported in previous DFT studies^12,34,36^. We also compare the diffusion barrier for a “cage-jump”, i.e., the O migration between two octahedral sites which are nearest neighbors to a Fe vacancy. The height of the barrier obtained with ACE is 0.56 eV, which agrees very well with 0.59 eV reported in a previous DFT study^36^.

It has been suggested^12^ that it is necessary to include the influence of Fe vacancies in order to explain a rather slow diffusion of O in BCC Fe observed experimentally. Both DFT calculations^34^ and the present ACE potential show that the proximity to a Fe vacancy indeed raises the energy barrier for O interstitial diffusion. Furthermore, since O is strongly bonded by the Fe vacancy (see Fig. 4b), the vacancy acts as an effective trapping site that influences the macroscopic diffusion, depending on the concentration of both species and ambient conditions (temperature and oxygen activity or pressure).

The formation energies of vacancies and interstitials in both Fe_3_O_4_ and Fe_2_O_3_ as a function of the oxygen chemical potential are presented in Fig. 3b, c, respectively. As can be seen in the experimental phase diagram of Fig. 1a, Fe_3_O_4_ is slightly off-stoichiometric at high temperatures, with a cation Fe deficiency^38^. For the two Fe lattice sites in Fe_3_O_4_, the vacancy formation energy is lower for the octahedral site than for the tetrahedral site, which was also reported experimentally^39^ and in previous DFT studies. The energy difference between the two sites predicted by ACE is 1.74 eV.

For interstitials in Fe_3_O_4_, there are three inequivalent sites: two tetrahedral and one octahedral with respect to the oxygen FCC sublattice^40^. For each site, the interstitial formation energy was evaluated for both Fe (in both spin configurations) and O. Only the results for the most favorable sites are shown in Fig. 3. According to the ACE potential, the open tetrahedral site is the most stable for the Fe interstitial, followed by the octahedral site with an energy difference of 0.52 eV. The second tetrahedral site is very small, and the Fe interstitial energy for this site is, therefore, very high. We also investigated the influence of the spin state of the Fe interstitial. The predicted energy difference between the two spin states is very small, but the configurations with Fe interstitial having a spin opposite to the surrounding Fe atoms were found to be more stable than those with the same spin directions. For O interstitials, the open tetrahedral site is also the most stable according to ACE, but the octahedral site has an energy higher by only 0.14 eV.

For Fe_2_O_3_, we report a higher formation energy of Fe vacancy than for Fe_3_O_4_. In Fe_2_O_3_, the most favorable site for both Fe and O interstitial atoms is in the octahedral position with respect to the HCP O sublattice, which was also reported in previous DFT studies^41^.

**Fig. 5:**
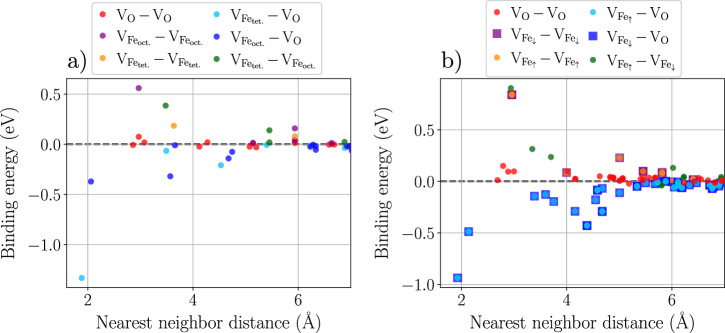
Vacancy binding energies in iron oxides. Binding energies between two vacancies in (**a**) Fe_3_O_4_ for all combinations of O (V_O_), tetrahedral (${{\rm{V}}}_{{{\rm{Fe}}}_{{\rm{tet.}}}}$) and octahedral (${{\rm{V}}}_{{{\rm{Fe}}}_{{\rm{oct.}}}}$) Fe vacancies, and (**b**) Fe_2_O_3_ for all combinations of O, spin up (${{\rm{V}}}_{{{\rm{Fe}}}_{\uparrow }}$) and spin down (${{\rm{V}}}_{{{\rm{Fe}}}_{\downarrow }}$) Fe vacancies, both obtained using the ACE potential. In (**b**), different symbols are used to avoid overlapping of configurations equivalent to spin inversion symmetry.

Figure 5 shows binding energies of different di-vacancies for pairs of V_Fe_–V_Fe_, V_Fe_–V_O,_ and V_O_–V_O_ in Fe_3_O_4_ and Fe_2_O_3_. In both oxides, only pairs composed of one Fe and one O vacancy have negative binding energies and only at short distances, indicating their tendency to bind and subsequently form clusters.

**Fig. 6:**
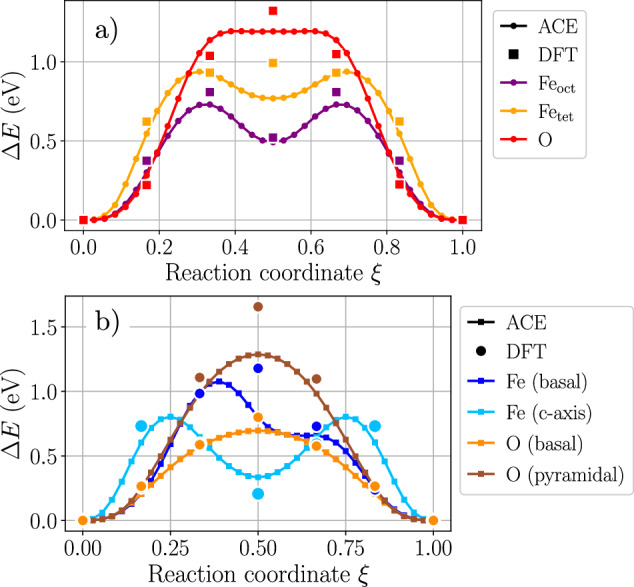
Vacancy diffusion barriers in iron oxides. Migration barriers of Fe and O vacancies in (**a**) Fe_3_O_4_ and in (**b**) *α*-Fe_2_O_3_. For Fe_3_O_4_, both tetrahedral and octahedral Fe vacancies are considered, both moving in a {100} plane. For Fe_2_O_3_, Fe and O atoms moving in different planes of the hexagonal lattice are considered: in the basal plane for both Fe and O, in the pyramidal plane for O, and along the *c* = [0001] axis for Fe. Large symbols correspond to DFT data computed in this work, all other symbols show the energy of each NEB image along the path predicted by the ACE potential.

Energy profiles along the vacancy migration pathways are presented for Fe_3_O_4_ and Fe_2_O_3_ in Fig. 6. Since the migration of interstitials is more complex and might involve spin flips as the cation moves through the material^42^, it is not discussed here.

In Fe_3_O_4_, the two types of Fe vacancies (tetrahedral and octahedral) have different diffusion barriers, with the energy barrier for octahedral Fe vacancies being lower. The results thus indicate that the vacancy-mediated diffusion of Fe in Fe_3_O_4_ is mainly due to the motion of octahedral vacancies, in line with experimental observations^39^.

For Fe_2_O_3_, Fe and O vacancies can diffuse along different paths: in the basal planes, the pyramidal planes, and along the *c* = [0001] axis of the underlying hexagonal O sublattice. For Fe vacancies, ACE predicts an easier motion along the *c* axis, with a slightly higher energy barrier in the basal planes. For O vacancies, ACE predicts an easier motion in the basal than in the pyramidal plane. The predicted barriers and their hierarchy are in good agreement with recent DFT + *U* results^32^, where different charge states were considered for each defect.

From the presented diffusion barriers, one can see that oxygen vacancies can move more easily in Fe_2_O_3_ than in Fe_3_O_4_. Additionally, Fe vacancies have a lower migration barrier than O in Fe_3_O_4_, while the barriers for the two species have similar heights in Fe_2_O_3_. While this can be linked to the transport properties of both species in the two oxides, it does not represent the full picture of diffusion, which must also account for the equilibrium concentration of defects. For instance, assuming the vacancy-mediated diffusion of both Fe and O in Fe_2_O_3_ to be solely described by the migration paths presented in Fig. 6b, Fe and O should diffuse at a comparable rate. However, as can be seen in Fig. 3c, the equilibrium concentration of defects depends strongly on the oxygen chemical potential. In the O-rich region, the formation energy of the O vacancy is significantly higher than that of the Fe vacancy, resulting in slower apparent O diffusivity due to the comparatively low concentration of the O vacancies. The situation will be reversed in the O-poor region where faster Fe diffusivity shall be observed. The full computation of the diffusion coefficients of Fe and O is however not within the scope of the present work and will be discussed in a separate study.

The transport properties of Fe and O in various compounds spanning the whole range of oxygen concentrations from pure Fe to the stable iron oxides is of utmost importance for understanding the mechanisms of oxidation and reduction of iron and its oxides^43^. Both are envisioned as key intertwined processes in the production of carbon-free energy from combustion of iron/iron oxides particles and the purification of iron through hydrogen-based direct reduction of iron oxides.

### Applications

**Fig. 7:**
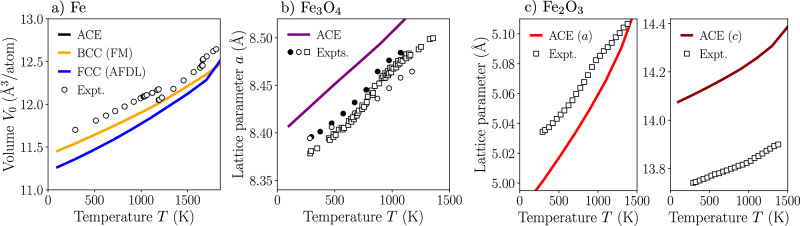
Thermal expansion of iron and iron oxides. Lattice thermal expansion computed using the ACE potential during molecular dynamics simulations at constant zero pressure for **a** pure Fe (BCC and FCC), **b** Fe_3_O_4_, and **c** Fe_2_O_3_. Results are compared to experimental data taken from ref. ^55^ for Fe, from ref. ^61^ (circles) and ref. ^62^ (squares) for Fe_3_O_4_, and from refs. ^17,63^ for Fe_2_O_3_. For Fe_2_O_3_, the two *a* (left) and *c* (right) lattice parameters of the hexagonal cell are presented.

In view of the targeted applications, it is also important to ensure that the ACE potential is able to capture finite temperature properties of the materials of interest. As in the previous sections, we focus on pure Fe and the two stable iron oxides, Fe_3_O_4_ and Fe_2_O_3_. The lattice expansion of these three prototypes, obtained by NPT molecular dynamics simulations under zero pressure, is presented in Fig. 7. The magnetic order in each material is kept fixed to that at the 0 K ground-state, namely FM and AFDL (antiferromagnetic double layer) for BCC and FCC Fe, respectively, ferrimagnetic for Fe_3_O_4_, and AF for Fe_2_O_3_ (see “Methods” for details).

Comparing to experimental data, we note that the potential is able to accurately describe the lattice expansion of pure Fe (Fig. 7a), the underestimation predicted by ACE being caused by the overbinding of the GGA-PBE functional. As for Fe_3_O_4_ and Fe_2_O_3_ (Fig. 7b, c, respectively), we note that ACE captures well the trends in the increase of lattice parameter for both oxides compared to experimental data. For Fe_2_O_3_, the potential somewhat overestimates the *c* lattice parameter of the hexagonal cell, but still reproduces the sharp increase observed experimentally. The slight changes in slope visible in the experimental data correspond to the Néel transition temperature from ordered magnetic states to a disordered PM state (around 950 K for Fe_3_O_4_ and Fe_2_O_3_^1^). This is not accounted for in the ACE results, since the magnetic orders were kept fixed during the simulations.

ACE predicts BCC Fe to melt at approximately 2250 K when the magnetic order is kept fixed to the FM state, which is an overestimation with respect to the experimental value of about 1800 K^23^. As for Fe_3_O_4_ and Fe_2_O_3_, they are predicted to melt at approximately 1750 K and 1650 K, respectively, which compares rather well with the experimental values of 1870 K and 1735 K^23^. It is also worth noting that the melting point of these two materials constitutes the major identified caveat of the ABOP2019 potential, predicting Fe_3_O_4_ and Fe_2_O_3_ to melt already at room temperature.

This section presents a series of stringent tests designed to assess the robustness of the ACE potential for configurations far from equilibrium and its ability to describe the thermodynamic properties of the Fe-O system through annealing simulations. Starting from an initially random periodic structure containing a given concentration of oxygen *x*_O_, the system is annealed at constant temperature and pressure during an NPT molecular dynamics run. Additionally, Fe atoms are allowed to change their spin state (Fe_*↑*_, Fe_*↓*_, Fe_0_) via semi-grand canonical Monte Carlo swaps (see “Methods” for details).

**Fig. 8:**
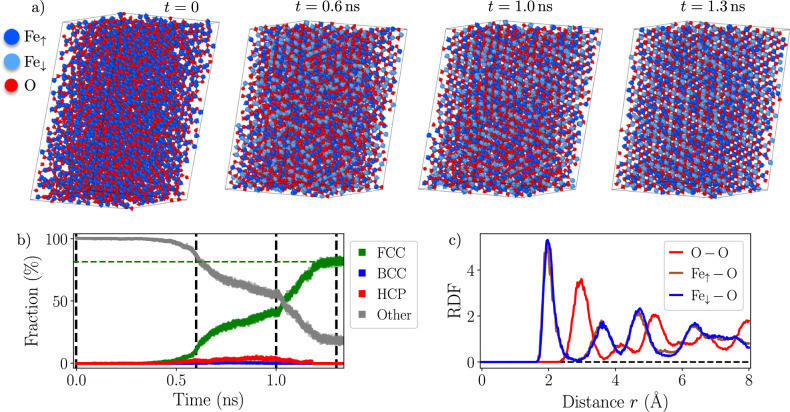
Annealing of random Fe-O structures. **a** Snapshots of an annealing MD-MC simulation of an initially random structure comprising 4480 atoms and a fixed oxygen concentration *x*_O_ = 55%. **b** Evolution of the symmetry of the underlying oxygen sublattice as a function of simulation time obtained using a common neighbor analysis algorithm as implemented in the OVITO software^64^. The times of the snapshots shown in (**a**) are marked by vertical lines. **c** Radial distribution function (RDF) for pairs of Fe_*↑*_−O (brown), Fe_*↓*_−O (purple) and O−O (red) after annealing the system for 1.3 ns.

In the example presented in Fig. 8, the structure consists of 4480 atoms with *x*_O_ = 55% annealed at 800 K under zero applied pressure. In a short time, the system is able to escape from the highly non-equilibrium starting configuration and starts to order. This test not only demonstrates the robustness of the potential, but also its ability to describe high-energy structures which can occur, for instance, in the liquid phase. During these simulations, we kept track of the extrapolation grade *γ*^44^ to evaluate uncertainty and to monitor whether the potential starts to extrapolate. Apart from a few initial iterations, the potential remains reliable with *γ* ≤ 1 throughout the whole simulation.

According to the experimental phase diagram, at the oxygen concentration of 55 % and temperature of 800 K, the equilibrium structure should consist of a mixture of wüstite (Fe_1−*x*_O) and Fe_3_O_4_. To determine the nature of the system after annealing, a simple descriptor to discriminate between different oxide structures is the symmetry of the underlying oxygen sublattice. Indeed, for wüstite and Fe_3_O_4_, the oxygen atoms arrange on a FCC lattice, while for Fe_2_O_3_, the oxygen sublattice is HCP^1^. Under the above conditions, we thus expect an oxygen FCC sublattice to form. As presented in Fig. 8b, the fraction of O atoms with an FCC environment gradually increases as the system is annealed, showing the system to anneal towards a mixture of Fe_1−*x*_O and Fe_3_O_4_.

The radial distribution functions for Fe-O pairs, plotted in Fig. 8c, show a peak at around 2 Å corresponding to the nearest neighbor distance between Fe and O atoms. This is close to the average value between 2.1 Å in wüstite and 1.9 Å/2.1 Å in Fe_3_O_4_ (for tetrahedrally and octahedrally coordinated Fe atoms respectively) under the same conditions of temperature and pressure. These observations demonstrate the ability of the ACE potential to describe a wide range of environments in terms of both oxygen concentration and atomic structures, while accurately capturing the thermodynamic properties of the Fe-O system.

**Fig. 9:**
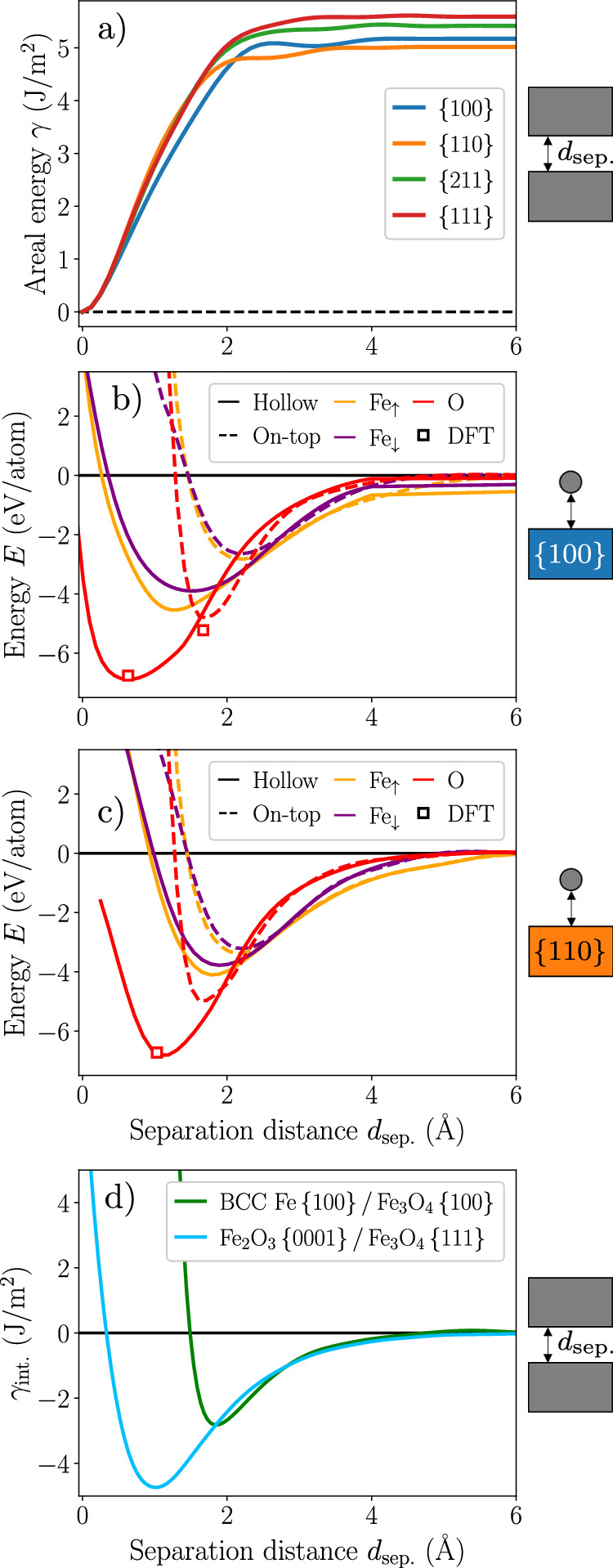
Decohesion in various environments. **a** Rigid decohesion of various surface facets of BCC Fe. Adatom detachment on a **b** {100} and **c** {110} surface of BCC Fe. DFT references for O adsorption are taken from ref. ^46^. **d** Rigid decohesion of BCC Fe/Fe_3_O_4_ (green solid line) and Fe_2_O_3_/Fe_3_O_4_ (blue solid line) coherent interfaces.

We present in Fig. 9 several decohesion scenarios involving different types of bonds and bonding environments (surfaces, adatoms and mixed interfaces). These pose stringent tests for any potential and several MLIPs fail to predict reasonable decohesion curves, compared to more simple and physically justified interatomic potentials^26^. These tests also have practical applications, for instance in the context of fracture, surface adsorption and interface cohesion in the cases examined here. We stress that none of these tests were explicitly included in the training database, except the equilibrium configuration of the Fe/Fe_3_O_4_ interface discussed below.

The rigid decohesions of the four low-index surfaces of BCC Fe ({100}, {110}, {211} and {111}) presented in Fig. 9a show a smooth behavior, converging to twice the surface energy of each facet (see Supplementary Materials for comparison). The {110} surface has the lowest energy, closely followed by {100}, as also reported in previous DFT studies^30,45^. Now considering adatom adsorption on BCC Fe surfaces (see Fig. 9b, c for {100} and {110} facets, respectively), the adhesion profiles at the high-symmetry hollow and on-top sites as a function of the separation distance to the surface show a smooth behavior for all atomic species (Fe, both spin up and down, and O). Most particularly, the adsorption energy of an O atom on the two surfaces of BCC Fe predicted by ACE agree very well with previous DFT studies on both {100} and {110} surfaces^46,47^. This property has been considered as one of the benchmarks for assessing the accuracy of various ReaxFF potentials in a recent work by Thijs et al.^4^, where the tested parameterizations yielded a considerable spread in the predicted adsorption energies compared to DFT.

We finally focus on bond-breaking environments at mixed coherent interfaces, i.e., interfaces between pure Fe and its oxides, or between different iron oxides. Such interfaces are fundamental in the understanding of dynamic processes such as hydrogen reduction, and more generally bulk phase transformations, involving coexistence of multiple phases^48^. Modeling of these complex structures requires an accurate description of bulk environments in both materials, but also highly distorted structures found at the very interface. We considered two different interfaces: BCC Fe/Fe_3_O_4_ and Fe_3_O_4_/Fe_2_O_3_.

The orientation relationships of the two interfaces were both determined experimentally in previous works. For the BCC Fe/Fe_3_O_4_ interface, it corresponds to Fe[001]_*z*_∥ Fe_3_O_4_[001]_*z*_ and Fe[100]_*x*_∥ Fe_3_O_4_[110]_*x*_^49^. For the Fe_3_O_4_/Fe_2_O_3_ interface, the orientation relationship corresponds to Fe_3_O_4_[111]_*z*_∥ Fe_2_O_3_[0001]_*z*_ and Fe_3_O_4_[$11{\bar{2}}$]_*x*_∥ Fe_2_O_3_[$11{\bar{2}}0$]_*x*_^48^. In the BCC Fe/Fe_3_O_4_ interface, the relative position of the two slabs yielding the most stable configuration of the interface corresponds to the Fe atoms of the outer Fe_3_O_4_{100} surfaces matching the hollow sites of the BCC Fe{100} surfaces^50^. For the Fe_3_O_4_/Fe_2_O_3_ interface, the relative position of the two slabs corresponds to the position in which the two O sublattices of Fe_3_O_4_ (FCC) and Fe_2_O_3_ (HCP) match such that the outermost layers of Fe_3_O_4_ coincides with the HCP lattice of Fe_2_O_3_^48^. More details about the setup and the geometry of the interface configurations are given in Methods.

The decohesion curves giving the work of adhesion *γ*_int._ of the interfaces are presented in Fig. 9d, showing a smooth variation as a function of the separation distance between the two slabs. In a previous work focusing on the Fe/Fe_3_O_4_ interface^50^, we reported an adhesion energy and equilibrium separation distance of −2.96 J/m^2^ and 1.9;Å, respectively, within DFT, which agree very well with the −2.82 J/m^2^ and 1.8 Å values predicted by the ACE potential. The adhesion of the Fe_3_O_4_/Fe_2_O_3_ interface is predicted to be stronger than for the Fe/Fe_3_O_4_ interface, with a shorter separation distance of 1.00 Å. This is due to the strong binding energy obtained from merging the O sublattices of the two oxides. Additionally, since most of the previous interatomic potentials fitted for the Fe-O cannot accurately describe both pure Fe and every oxide structure, we demonstrate the present ACE potential to be currently the only one able to study these extended defects.

## Discussion

We developed an accurate and transferable ACE interatomic potential that is able to describe the complexity and variety encountered within the Fe-O system across the whole range of oxygen concentrations. Based on an extensive DFT-computed training set, which encompasses structures spanning a wide range of structural and chemical environments, we demonstrate the robustness of the fitted ACE potential on a broad variety of properties that are relevant for iron and its oxides. The focus was put mainly on thermodynamics, point defect properties, but also finite temperature behavior and structures far from equilibrium. For all these cases, the ACE predictions agree closely with DFT reference and experimental data. Given the extent of the DFT training set used for the ACE parameterization, the potential is expected to perform reasonably well also for the liquid phases of the Fe-O system.

The limitations of the present ACE potential are mainly related to the DFT training data, as any MLIP can only be as reliable as the data used for its training. In the case of the Fe-O system, choosing the most suitable training data presents a challenge, since no DFT flavor is able to describe both metallic Fe and its insulating oxides equally well. Consequences of our choice to use the standard GGA-PBE functional are reflected by the results presented in this work. For instance, the experimentally reported high pressure and temperature phase^24^ FeO_2_ (cf. Fig. 1b) is predicted as not being stable by DFT + *U*, in agreement with experiments, but not GGA-PBE. Furthermore, stoichiometric wüstite (FeO) is not thermodynamically stable at any temperature under zero pressure, but dissociates into a mixture of Fe and Fe_3_O_4_. However, for the present ACE model this oxide remains stable at finite pressures. Nevertheless, we believe that the GGA-PBE functional presents the best compromise when it comes to a reliable description of structural properties. Since the present ACE potential is aimed mainly at studies of thermodynamic behavior, diffusion and defect properties, the reliable description of the structure-energy relationship is of utmost importance.

The explicit inclusion of magnetism in the Ising-like manner allows to perform large-scale simulations where both magnetic and atomic degrees of freedom can evolve dynamically at a reasonable computational cost. Moreover, a discretization of magnetic moments allows us to avoid the expensive sampling of magnetic moment magnitudes, which is necessary for more flexible models^30^, thus enhancing data efficiency. Even though the current model does not allow for accurate modeling of magnetic excitations, it is able to capture the essential features of magnetism for both pure Fe and its oxides. Similarly, the explicit account of charges might also be of great importance to capture long-range interactions in the oxides, which is the subject of future work.

Overall, we believe the presented ACE model is a state-of-the-art interatomic potential that can be applied in complex large-scale atomistic simulations of the technologically extremely important Fe-O system. Further extension of the model to the ternary system Fe-O-H is currently in progress.

## Methods

### DFT calculations

All DFT calculations presented in the work were carried out using the VASP package^51^. As discussed in the main text, the GGA-PBE xc-functional^13^ was used in all calculations. Projector-augmented wave (PAW) pseudo-potentials^52^ were used to describe Fe and O atoms, including 8 and 6 valence electrons respectively. We use a plane-wave energy cutoff of 500 eV for all DFT calculations, with a *Γ*-centered *k*-point mesh of density 0.02 2 *π* Å^−1^. Magnetism is included in all calculations in its collinear approximation within spin-polarized DFT. Atomic relaxations were considered converged when the maximum component of the remaining forces on all atoms was less than 5 meV/Å.

### Diffusion barriers

Vacancy and interstitial migration barriers (see Table 2 and Fig. 6) were computed using the nudged elastic band (NEB) method considering five intermediate images linked by a spring constant of 5 meV/Å. Single vacancy and oxygen migration barriers in BCC Fe presented in Table 2 were computed in a 3 × 3 × 3 supercell containing 54 atoms. The vacancy migration barriers in Fe_3_O_4_ presented in Fig. 6a were computed in the conventional cubic cell containing 56 atoms. Vacancy migration barriers in Fe_2_O_3_ presented in Fig. 6b were computed in a rhombohedral cell having axes *x*∥[$1{\bar{1}}00$], *y*∥[$11{\bar{2}}0$] and *z*∥[0001] containing 120 atoms.

### MD-MC simulations

Hybrid molecular dynamics-Monte Carlo (MD-MC) simulations presented in this work were performed using the LAMMPS code^53^. The MD-MC simulations were employed to equilibrate the magnetic configuration of a given structure. To do so, we allow Fe atoms to swap between the two spin up and spin down species handled by the ACE potential. This is performed within a semi-grand canonical MC swap algorithm as implemented in LAMMPS, where the proportion of spin up and down Fe atoms is allowed to change during the simulation^54^. The chemical potential difference between the two spin states, while performing the swapping attempts, is set to zero, and the temperature for the MC swaps is set to the temperature of the ongoing MD. A timestep of 1 fs was used for all MD simulations performed in this work. We performed 100 MC swap attempts every 10 steps of the MD. The initial structure used in the annealing simulation presented in Fig. 8 was generated using the BUILDCELL code from the AIRSS suite^21^.

### Thermal expansion

The lattice expansion dependencies for Fe (BCC and FCC phases), Fe_3_O_4_ and Fe_2_O_3_ presented in Fig. 7 were obtained by averaging the lattice parameters of the materials during NPT molecular dynamics runs under zero pressure. The duration of the MD runs was 100 ps after an initial equilibration for 10 ps. The supercells used for BCC and FCC Fe contained 2000 and 2304 atoms, respectively. For Fe_3_O_4_ and Fe_2_O_3_, the supercells contained 1512 and 1800 atoms, respectively. During the simulations, the magnetic order of each material was kept fixed to its 0 K ground-state as defined in the main text.

Melting points were estimated as a temperature where the variation of volume as a function of temperature changes slope abruptly following the NPT procedure described above.

### Defect formation energies

For point defects formation energies *E*_*f*_ as a function of the relative oxygen chemical potential Δ*μ*_O_, the following equation was used

1 $${E}_{f}={E}_{{\rm{def.}}}-{E}_{{\rm{bulk}}}\pm ({E}_{{\rm{ref.}}}^{i}+\Delta {\mu }_{i}),$$

where *E*_def._ and *E*_bulk_ are the total energies of the simulation cell containing a point defect and the perfect bulk crystal respectively, and ${\mu }_{i}={E}_{{\rm{ref.}}}^{i}+\Delta {\mu }_{i}$ is the chemical potential of species *i*. The plus (minus) sign applies for vacancies (interstitials).

We impose Fe and O atoms to be in equilibrium with the material considered, enforcing the relation ${E}_{{{\rm{Fe}}}_{3}{{\rm{O}}}_{4}}=3\,{\mu }_{{\rm{Fe}}}+4\,{\mu }_{{\rm{O}}}$ in Fe_3_O_4_ and resulting in ${\mu }_{{\rm{Fe}}}=1/3[{E}_{{{\rm{Fe}}}_{3}{{\rm{O}}}_{4}}-4\,{\mu }_{{\rm{O}}}]$. Similarly in Fe_2_O_3_, we have ${E}_{{{\rm{Fe}}}_{2}{{\rm{O}}}_{3}}=2\,{\mu }_{{\rm{Fe}}}+3\,{\mu }_{{\rm{O}}}$, and thus ${\mu }_{{\rm{Fe}}}=1/2[{E}_{{{\rm{Fe}}}_{2}{{\rm{O}}}_{3}}-3\,{\mu }_{{\rm{O}}}]$. These relations then allow to plot the formation energies of point defects as a function of the oxygen chemical potential only, as presented in Fig. 3. The convergence of the formation energies with respect to the size of the simulation cell was checked for each prototype material presented.

### Interface structures

For the two interfaces presented in Fig. 9d, we used slab structures periodic in the two in-plane directions with open surfaces and a 20 Å-thick vacuum layer in the direction normal to the interface plane. The thickness of the two slabs in the direction normal to the interface was set to approximately 20 Å. The outermost atomic layers in the direction normal to the interface were kept fixed during all subsequent calculations.

Before evaluating the interfacial energies of the two cases, we first searched for the relative position of the two slabs that minimized the total energy of the interface structures. The distance between the two slabs in the direction normal to the interface plane is then varied, and the interfacial energy *γ*_int._ is evaluated as

2 $${\gamma }_{{\rm{int.}}}=\left[{E}^{{\rm{tot.}}}-\left({E}^{{\rm{slab}}1}+{E}^{{\rm{slab}}2}\right)\right]/S,$$

with *E*^tot.^ the total energy of the simulation cell containing the two slabs of energies *E*^slab 1^ and *E*^slab 2^, and *S* the surface of the interface structure.

## Supplementary information

Revised Supplementary Information

## Data Availability

The files for the ACE Fe-O potential, including sample scripts for running different simulations, are available at 10.5281/zenodo.14499961. We also provide in Supplementary Note 5 a detailed technical description of the implementation of the potential and how to run different types of simulations using the LAMMPS code.
