# Supplementary material for: Development of an atomic cluster expansion potential for iron and its oxides
Source: NPJ Comput Mater. 2025 Mar 26;11(1):81. doi: 10.1038/s41524-025-01574-w (PMC11946894; doi:10.1038/s41524-025-01574-w)
Supplement: Supplementary file 1 — Revised Supplementary Information [file 41524_2025_1574_MOESM1_ESM.pdf]

Supplementary Information: Development of an Atomic Cluster Expansion potential for iron and its oxides

Baptiste Bienvenu,* Mira Todorova,† Jörg Neugebauer,‡ and Dierk Raabe§
Max Planck Institute for Sustainable Materials,
Max-Planck-Straße 1, 40237 Düsseldorf.

Matous Mrovec, Yury Lysogorskiy, and Ralf Drautz
Interdisciplinary Centre for Advanced Materials Simulations,
Ruhr Universität Bochum, 44780 Bochum.

*Electronic address: b.bienvenu@mpie.de
†Electronic address: m.todorova@mpie.de
‡Electronic address: j.neugebauer@mpie.de
§Electronic address: d.raabe@mpie.de

Supplementary Note 1: Additional validation on pure Fe

We present in this section extensive additional validation of the Fe-O ACE potential performed on pure Fe, including bulk and defect properties.

Bulk properties

Supplementary Tables I and II summarize elastic constants C_{ij} of BCC, FCC and HCP Fe. For each, we considered different magnetic orders, namely FM and NM for BCC, AFDL and NM for FCC, and AF and NM for HCP. When available or computed for this study, DFT references are included. Available experimental data are also presented. For the different crystal structures and magnetic orders considered here, we note a very good agreement between the ACE potential and reference data.

Supplementary Table I: Bulk properties (lattice parameter a_0, bulk modulus B_0 and elastic constants C_{ij}) of the FM and NM orders of BCC Fe, and defect formation energies (vacancy $E_f^{\text{vac.}}$, self-interstitial atom with various dumbbell configurations E_f^{dhkl}, and surface energies $\gamma^{\{hkl\}}$, where hkl denote the Miller indices of the crystallographic orientation) in the FM ground-state only.

	ACE	DFT	Expt.
Fe, BCC FM			
a_0 (Å)	2.84	2.83	2.86 [1]
B_0 (GPa)	165	191 [2]	170 [3]
C_{11} (GPa)	240	283 [2]	240 [3]
C_{12} (GPa)	127	145 [2]	136 [3]
C_{44} (GPa)	91	104 [2]	121 [3]
$E_f^{\text{vac.}}$ (eV)	2.1	2.2	2.0 ± 0.2 [4]
E_f^{d110} (eV)	4.6	4.9 [2]	4.7 - 5.0 [5]
E_f^{d100} (eV)	5.6	5.3 [2]	/
E_f^{d111} (eV)	4.9	4.9 [2]	/
$\gamma^{\{100\}}$ (J/m^2)	2.58	2.49	/
$\gamma^{\{110\}}$ (J/m^2)	2.52	2.42	/
$\gamma^{\{112\}}$ (J/m^2)	2.71	2.56	/
$\gamma^{\{111\}}$ (J/m^2)	2.79	2.69	/

	ACE	DFT	Expt.
Fe, BCC NM			
ΔE (eV/atom)	0.47	0.48	/
a_0 (Å)	2.75	2.75	/
B_0 (GPa)	280	270 [2]	/
C_{11} (GPa)	39	87 [2]	/
C_{12} (GPa)	400	361 [2]	/
C_{44} (GPa)	189	180 [2]	/

According to the ACE potential, FM FCC Fe is unstable, considering its two stable magnetic configurations, namely low-spin and high-spin. However, this is not the magnetic ground-state of FCC Fe, which is the antiferromagnetic double layer (AFDL), whose equilibrium properties are presented on the left column of Supplementary Table II, along with those of non-magnetic (NM) FCC Fe. As for the HCP phase of pure Fe, its equilibrium properties are presented on the right column of Supplementary Table II, for both the AF and NM magnetic orders.

Supplementary Table II: Same data as presented in Supplementary Table I for the BCC phase of Fe, but for the FCC (left) and HCP (right) phases, with the vacancy diffusion barrier $\Delta H^{\mathrm{vac.}}$.

	ACE	DFT	Expt.
Fe, FCC AFDL			
ΔE (eV/atom)	0.08	0.08	/
a_0 (Å)	3.55	3.55	/
B_0 (GPa)	148		146 [6]
C_{11} (GPa)	351	/	/
C_{12} (GPa)	26	/	/
C_{44} (GPa)	87	/	/
$E_f^{\mathrm{vac.}}$ (eV)	1.79	1.82 [7]	/
$\Delta H^{\mathrm{vac.}}$ (eV)	0.71	0.74	/
Fe, FCC NM			
ΔE (eV/atom)	0.16	0.17	/
a_0 (Å)	3.45	3.45 [2]	/
B_0 (GPa)	288	281 [2]	146 [6]
C_{11} (GPa)	465	414 [2]	/
C_{12} (GPa)	199	214 [2]	/
C_{44} (GPa)	201	240 [2]	/
$E_f^{\mathrm{vac.}}$ (eV)	2.27	2.27 [7]	/

	ACE	DFT	Expt.
Fe, HCP AF			
ΔE (eV/atom)	0.06	0.06	/
V_0 (Å^3/atom)	10.54	10.55	/
B_0 (GPa)	195	199	/
C_{11} (GPa)	457	/	/
C_{12} (GPa)	84	/	/
C_{44} (GPa)	109	/	/
$E_f^{\mathrm{vac.}}$ (eV)	1.70	2.27	/
$\Delta H^{\mathrm{vac.}}$ (eV)	1.06	1.28	/
Fe, HCP NM			
ΔE (eV/atom)	0.09	0.10	/
V_0 (Å^3/atom)	10.14	10.18	
B_0 (GPa)	313	291	/
C_{11} (GPa)	545	/	/
C_{12} (GPa)	157	/	/
C_{44} (GPa)	180	/	/
$E_f^{\mathrm{vac.}}$ (eV)	2.07	2.45	/

We also present in Supplementary Figure 1 transformation paths between different crystal

structures for pure Fe with a FM order. Along these paths, the coordination, bond distances and angles change significantly, making them a sensitive test when assessing the robustness of any interatomic potential [8]. We again report a very good agreement between the DFT reference data and the results obtained with the ACE potential, demonstrating its robustness and accuracy when describing highly distorted atomic environments.

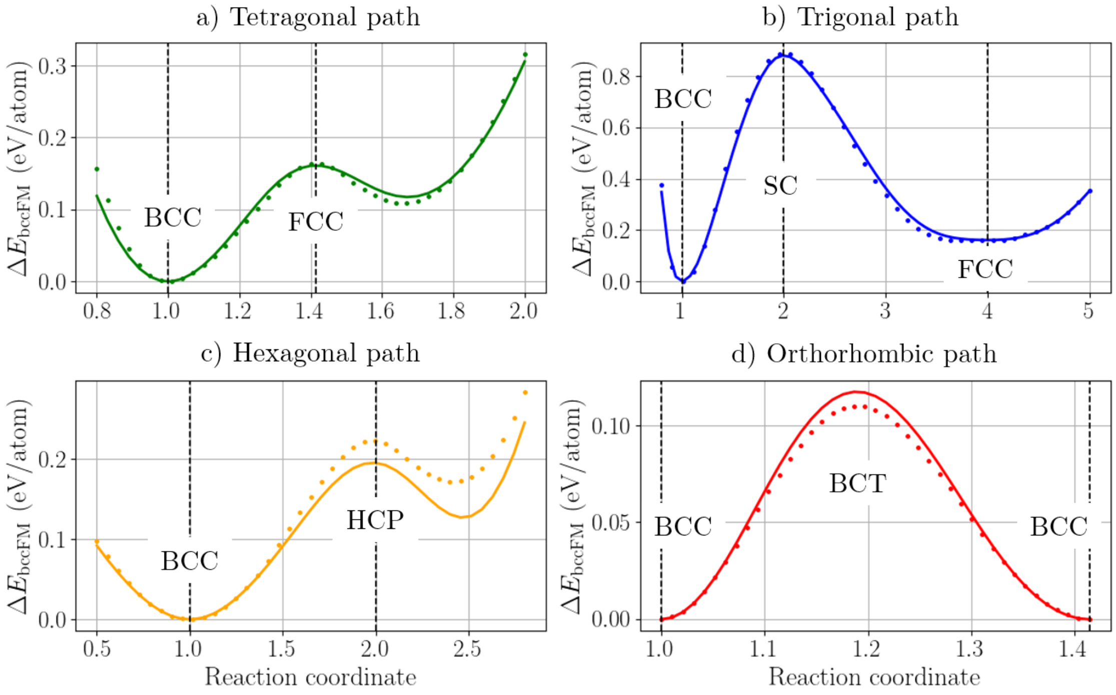

Supplementary Figure 1: Transformation paths of Fe (FM magnetic order): a) tetragonal (or Bain) path from BCC to FCC ; b) trigonal path from BCC to simple cubic (SC) to FCC ; c) hexagonal path from BCC to HCP ; d) orthorhombic path from BCC to body-centered tetragonal (BCT).

Finally, phonon spectra for the four BCC FM, FCC AFDL, HCP AF and $A15$ FM phases of pure Fe are presented in Supplementary Figure 2 and compared to results obtained using DFT. We note a very good agreement between the two methods, demonstrating that the ACE potential is also able to capture vibrational properties in a wide range of atomic configurations in the case of pure Fe.

Defect properties

Defect properties are presented only in the stable structures, which are BCC FM, FCC AFDL and NM, HCP AFII and NM. The stability of the different crystal structures are

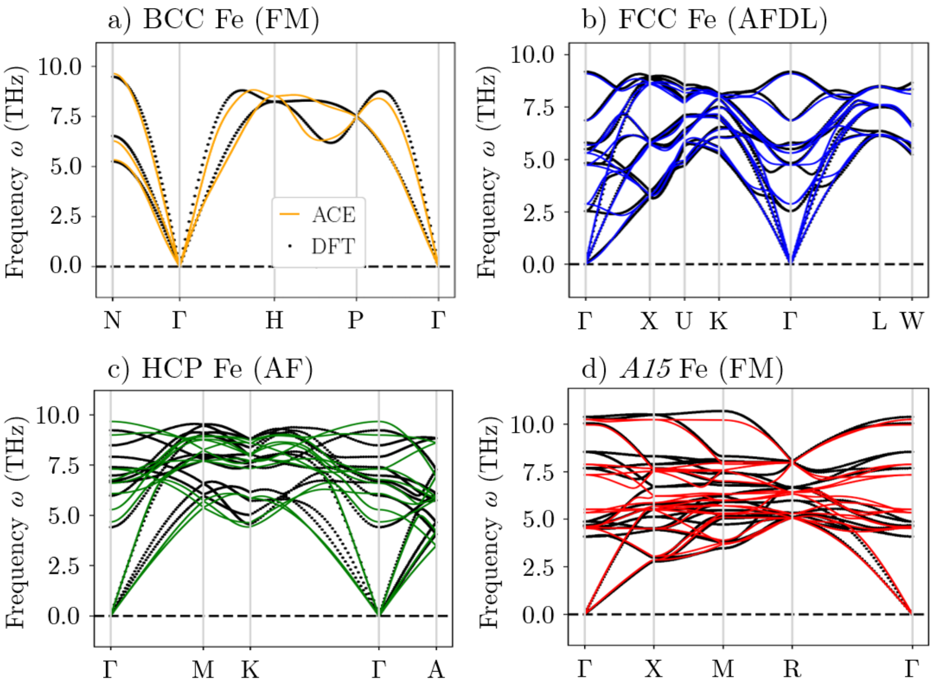

Supplementary Figure 2: Phonon spectrum for a) BCC Fe (FM magnetic order), b) FCC Fe (AFDL magnetic order), c) HCP Fe (AF magnetic order), and d) *A*15 Fe (FM magnetic order). DFT data (black dots) are compared to the results of the ACE potential (solid color lines).

assessed through the existence of imaginary phonon modes.

We present in Supplementary Tables I and II the vacancy formation energies for the different crystal structures of pure Fe. We note a very good agreement with available DFT references in the three phases of pure Fe, with a lower formation energy in the FCC and HCP phases than in the BCC phase. For the FM magnetic order of BCC Fe, we also compare in Supplementary Table I the surface energies of different crystal orientations predicted by ACE with DFT reference data, between which we note a very satisfactory agreement. In the same phase, we also computed the formation energy of self-interstitial Fe atoms having different dumbbell configurations (see Supplementary Table I). Values predicted by ACE are in very good agreement with previously reported DFT values, for instance reproducing the very close energies of the two $\langle 110 \rangle$ and $\langle 111 \rangle$ configurations.

We stress that some of the presented properties are usually difficult to accurately reproduce for interatomic potentials, for instance vacancy formation energies, self-interstitials and the hierarchy between different configurations, and the almost degeneracy between the two $\{100\}$ and $\{110\}$ surfaces of BCC FM.

We also further validate the present ACE potential on more complicated defects, namely dislocations. As a first step towards rationalizing the motion of dislocations in a crystal, the generalized stacking faults give interesting information on the ease to shear different crystallographic planes (assimilated to the glide planes of dislocations) by a given vector (assimilated to the Burgers vector of dislocations). These are presented in Supplementary Figure 3 in the two {110} and {112} planes of BCC Fe, which are the two main glide planes of dislocations in BCC metals.

Cuts along the ⟨111⟩ directions contained in the two planes are also presented in Supplementary Figure 3 c, where 1/2⟨111⟩ represent the main Burgers vector of dislocations in BCC crystals. We note that compared to DFT data, the ACE potential captures very well the shearing of the lattice in the two considered planes, also reproducing the asymmetry in the {112} planes (red line in Supplementary Figure 3 c).

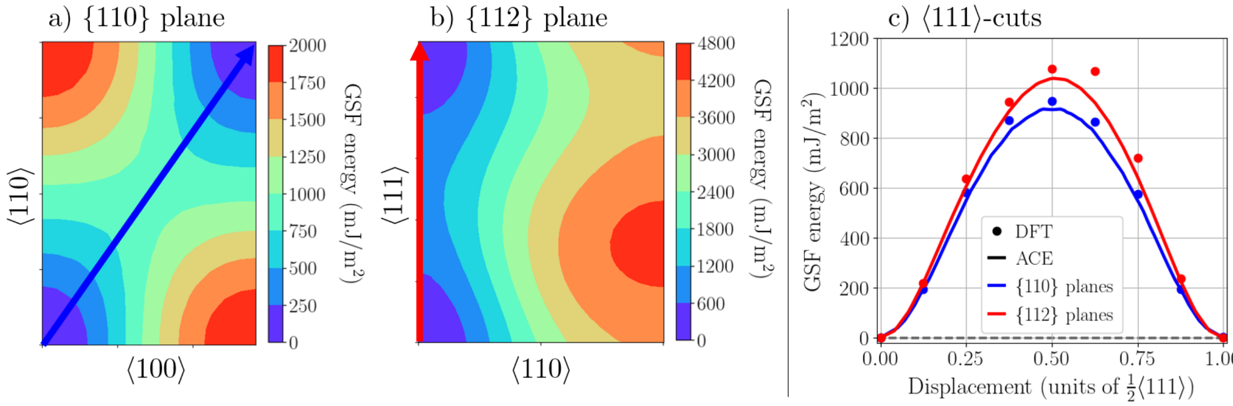

Supplementary Figure 3: Generalized stacking faults in a a) {110} and b) {112} plane of BCC Fe (FM magnetic order) obtained using the $ACE_{Fe\text{-}O}$ potential. c) Cuts of the γ-surfaces along a 1/2⟨111⟩ direction in {110} (blue) and {112} (red) planes.

We present in Supplementary Figure 4 the core structure and the Peierls energy barrier for the 1/2⟨111⟩ screw dislocation in BCC Fe (FM magnetic order). Plasticity of BCC metals is governed at low temperature by the motion of these 1/2⟨111⟩ screw dislocations through the crystal, due to friction they experience with the lattice. Thus, in order to be able to accurately describe the plastic deformation of BCC Fe, it is important that the ACE potential is also able to describe its core structure and the energy barrier associated with its motion, namely the Peierls barrier. We note that the Fe-O ACE potential accurately predicts the compact core structure for the 1/2⟨111⟩ screw dislocation, with a Peierls barrier

of very similar height compared to DFT reference. We also note that these properties are usually hard to correctly reproduce by classical EAM interatomic potentials [9].

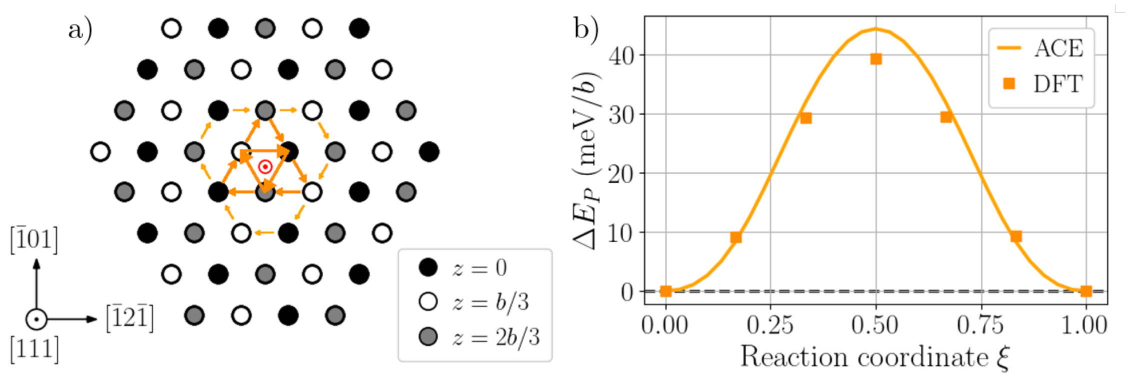

Supplementary Figure 4: a) Core structure of a $1/2\langle 111\rangle$ screw dislocation predicted by the ACE potential plotted as a differential displacement map along the [111] direction. b) Peierls barrier opposing glide of a $1/2\langle 111\rangle$ in a {110} plane between two adjacent equilibrium positions. DFT data is taken from Ref. [10].

Finally, we present in Supplementary Figure 5 the generalized stacking fault of FCC Fe in the (111) plane, cut along the $[11\bar{2}]$ direction. First of all, we note a very good agreement between the predictions of the ACE potential and DFT, also in agreement with a previous DFT study [11]. Looking at the line cut, the stable stacking fault (located at a shearing of $2/6\,[11\bar{2}]$) corresponds to a local HCP structure near the fault plane. The negative stable stacking fault energy obtained in FCC Fe with a NM order indicates that the HCP structure is more stable than FCC. This is not the case in the AF magnetic order, where the stable stacking fault has a positive energy, directly linked to the dissociation of dislocations in FCC Fe.

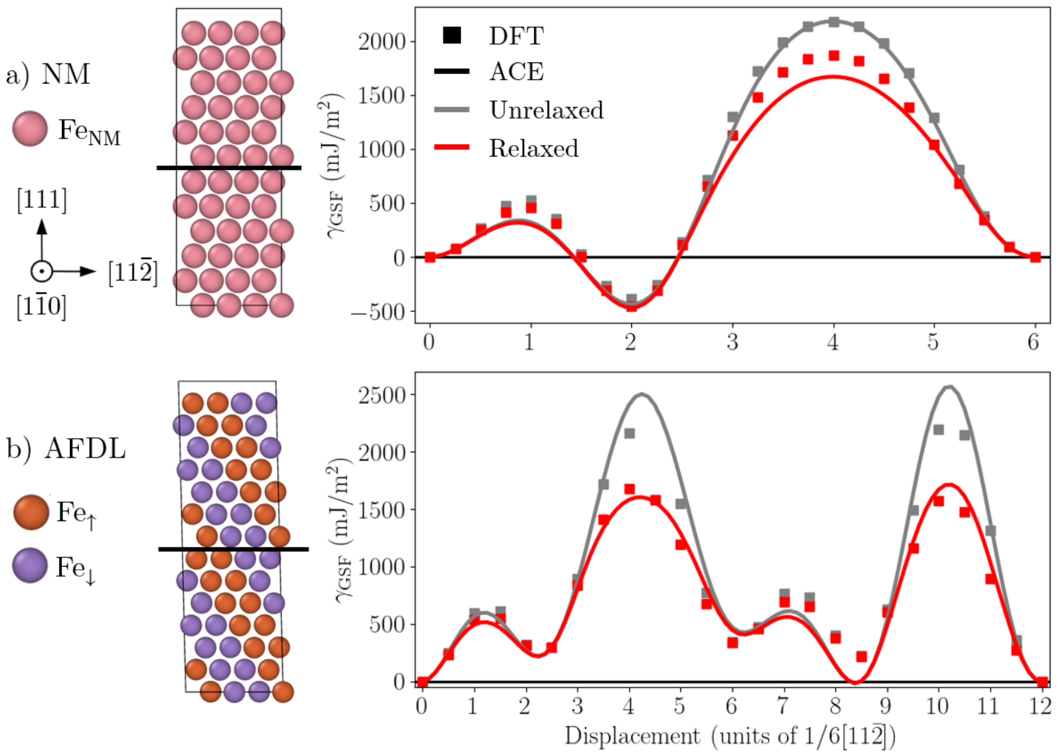

Supplementary Figure 5: Cut of the {111} γ-surface of FCC Fe along a ⟨112⟩ direction in the a) NM and b) AFDL magnetic states. The path for the AFDL structure is twice longer than the NM case due to the breaking of the periodicity of the magnetic order by a ⟨112⟩ shear in a {111} plane.

Supplementary Note 2: Additional validation on oxides

As for iron oxides, we present in Supplementary Figure 6 the phonon spectrum of Fe_3O_4 and Fe_2O_3, comparing the results obtained using ACE and DFT. We note a very satisfactory agreement between the two methods for both oxides, showing the ability of the ACE potential to also describe vibrational properties of iron oxides.

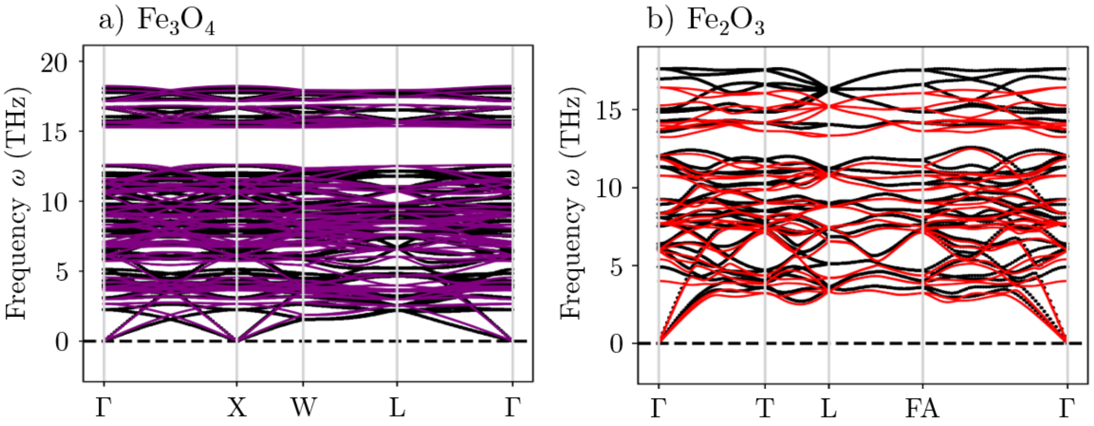

Supplementary Figure 6: Phonon spectrum for a) Fe_3O_4 (ferrimagnetic order) and b) Fe_2O_3 (AF magnetic order). DFT data (black dots) are compared to the results of the $ACE_{Fe\text{-}O}$ potential (solid color lines).

Supplementary Note 3: Effect of DFT xc-functional on bulk properties of Fe

As mentioned in the main text, we investigated the effect of using different DFT xc-functionals on the bulk properties of pure Fe. The results are presented in Supplementary Figure 7, considering GGA-PBE, different values for the U_{Fe} correction added on the $3d$ orbitals of Fe atoms, and the SCAN meta-GGA xc-functionals.

Comparing pure GGA-PBE results with DFT + U, we observe that increasing the values of the U_{Fe} correction gradually decreases the energy difference between the BCC FM and the FCC AFDL phases of pure Fe until the two phases have almost the same energy for $U_{\mathrm{Fe}} = 4\,\mathrm{eV}$ (see Fig. 7 d). Increasing U_{Fe} also raises the energy of all NM phases of Fe, regardless of the crystal structure. On the other side, the use of the SCAN xc-functional yields results in a rather good agreement with GGA-PBE for the two BCC and FCC phases. However, the energy of the HCP NM phase is predicted rather high, pushing the pressure at which BCC Fe would transform to HCP.

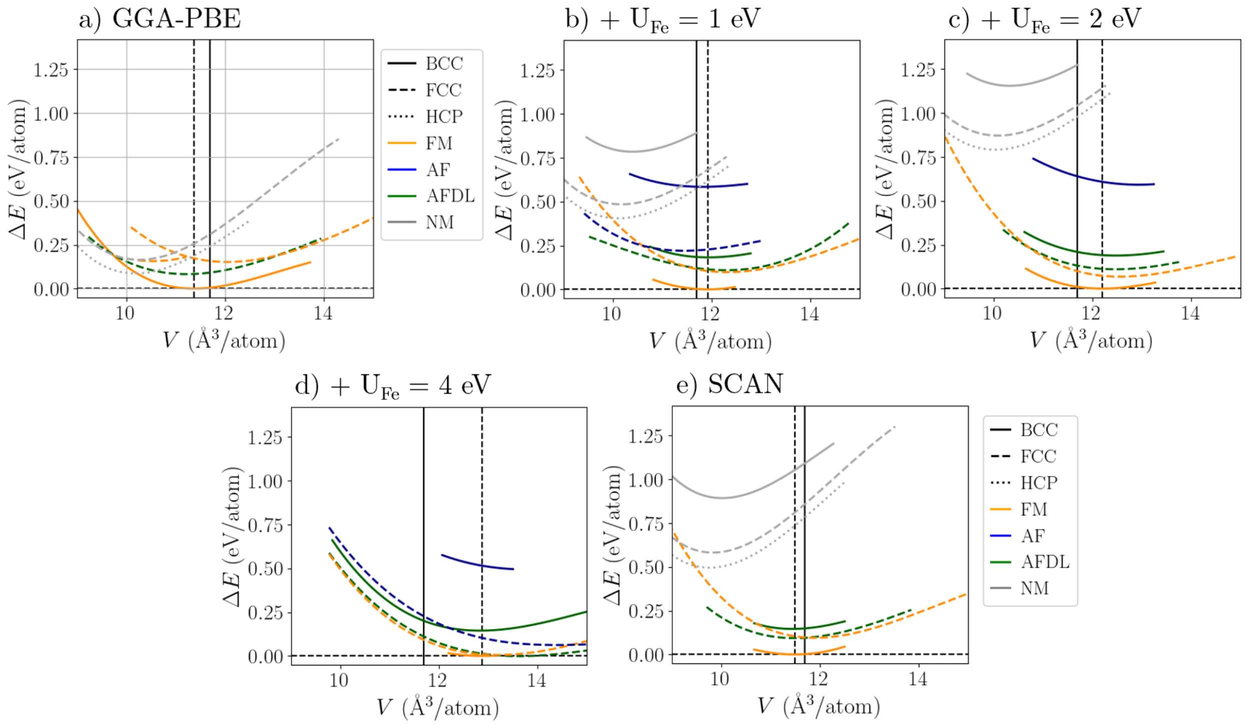

Supplementary Figure 7: Energy as a function of atomic volume for different crystal structures and magnetic orders of pure Fe using different DFT functionals: a) standard GGA-PBE, b) GGA-PBE+ $U_{\mathrm{Fe}} = 1\,\mathrm{eV}$, c) GGA-PBE + $U_{\mathrm{Fe}} = 2\,\mathrm{eV}$, d) GGA-PBE + $U_{\mathrm{Fe}} = 4\,\mathrm{eV}$, and e) SCAN.

Supplementary Note 4: Test cases for the spin equilibration approach

We present in this section test cases to benchmark the spin equilibration scheme described in the main text.

For this purpose, we rely on semi-grand canonical Monte Carlo swaps performed between the different magnetic Fe species handled by the ACE Fe-O potential, namely $Fe_{\uparrow}$ and $Fe_{\downarrow}$. All simulations are performed using the LAMMPS code, and its implementation of semi-grand canonical Monte Carlo swaps described in Ref. [12].

We benchmark this procedure considering the two Fe_3O_4 and Fe_2O_3 iron oxides, for which the magnetic ground-state in non-trivial (ferrimagnetic for Fe_3O_4, and antiferromagnetic for Fe_2O_3) to demonstrate its robustness on complex cases. For this purpose, we considered a supercell of bulk Fe_3O_4 containing a total of 7000 atoms (3000 of which being Fe atoms), and a supercell of Fe_2O_3 containing 7200 atoms (2880 of which being Fe atoms), both initialized with a completely randomized distribution of the Fe spins. We plot Fig. 8 the order parameter that describes the magnetic order of Fe_3O_4 in a and Fe_2O_3 in b as a function of the simulation time.

During the equilibration MD-MC run, the temperature of both MD and MC swap attempts between different Fe magnetic species is set to 800 K. For Fe_3O_4, the magnetic ground-state is ferrimagnetic, with Fe atoms sitting on the two tetrahedral and octahedral sub-lattices having opposite spin directions (see sketch of the unit cell above). For Fe_2O_3, the magnetic ground-state is antiferromagnetic, with a stacking of the atomic layers corresponding to Fe↑-Fe↑-O-Fe↓-Fe↓ along the [0001] axis (see sketch in Fig. 8).

After a few tens of picoseconds, the magnetic ground-state of both Fe_3O_4 and Fe_2O_3 is found by the annealing procedure, with the two Fe sub-lattices completely populated with Fe atoms of the same spin in each case.

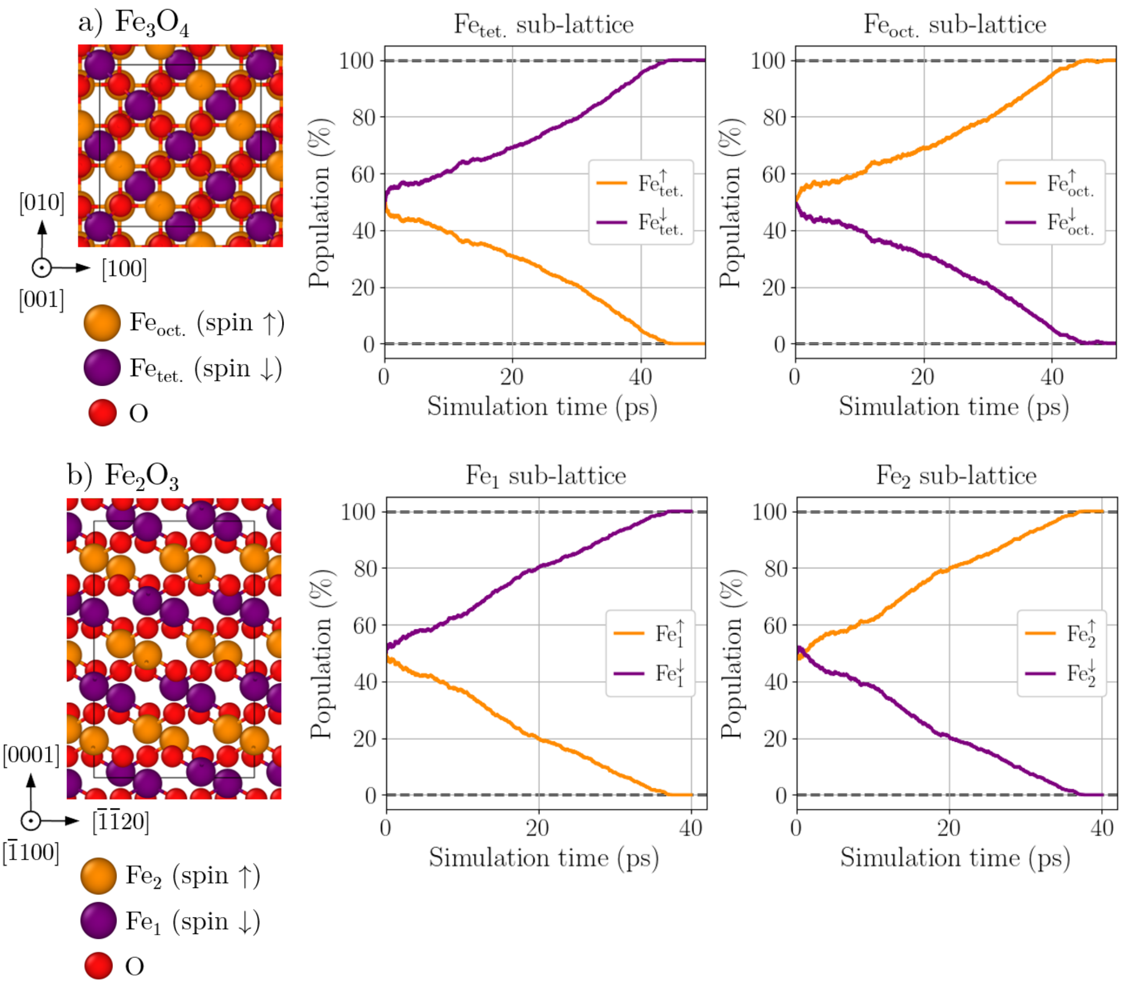

Supplementary Figure 8: Evolution of the Fe magnetic order of a supercell containing a) Fe_3O_4 and b) Fe_2O_3 upon annealing of the magnetic degrees of freedom during a hybrid MD-MC simulation described in the Methods section. The temperature of both the MD thermostat and the MC swapping attempts between Fe magnetic species is set to 800 K for both Fe_3O_4 and Fe_2O_3. For each oxide, the population of spin up and spin down Fe atoms is presented for the two Fe sub-lattices: $Fe_{tet.}$ and $Fe_{oct.}$ for Fe_3O_4, and Fe_1 and Fe_2 for Fe_2O_3, respectively (see structural models on the left for definition of the Fe sub-lattices).

Supplementary Note 5: Note on the use of the ACE Fe-O potential

In this section, we discuss some technical aspects of running simulations with the presented Fe-O ACE for potential users of the model. As stated in the Data Availability section of the main text, potential files and additional information (including example LAMMPS scripts) are available on the zenodo directory: 10.5281/zenodo.14499961

LAMMPS *installation and ACE support*

To install LAMMPS with support for ACE potentials, please follow the instructions given here: https://pacemaker.readthedocs.io/en/latest/

General information about the ACE Fe-O potential architecture

As described in the main text, the present ACE Fe-O potential explicitly accounts for magnetic degrees of freedom on Fe atoms with an Ising-like model, *i.e.* considering three different types of Fe atoms depending on the sign of their magnetic moments: $Fe_{\uparrow}$ for spin up, $Fe_{\downarrow}$ for spin down, and Fe_{NM} for non-magnetic or zero spin.

So that LAMMPS can run with the three difference types of Fe atoms, they were named after existing atomic species, namely "Fe" for $Fe_{\uparrow}$, "Mn" for $Fe_{\downarrow}$, and "Co" for Fe_{NM}. Thus one has to set the mass of the different types of Fe magnetic species to the atomic mass of Fe at the start of a given simulation to avoid LAMMPS assigning different masses to the different magnetic Fe species.

When initializing the system for further simulation, one needs to set the desired magnetic order beforehand by assigning different magnetic species to each atom of the structure. Otherwise, for instance if the magnetic ground state of the system is not known, one can rely on the annealing procedure described in Supplementary Note 4 above.

Molecular dynamics with on-the-fly spin equilibration

To perform MD runs with on-the-fly equilibration of the magnetic degrees of freedom, as discussed in the main text, we rely on an approach based on hybrid MD-MC simulations.

This approach uses on **fix atom/swap** implemented in LAMMPS [12], along with a conventional molecular dynamics run. In particular we allow swaps between the two $Fe_{\uparrow}$ and $Fe_{\downarrow}$ magnetic species to allow for equilibration of the magnetic order of the system. Every 10 MD steps, 100 swap attempts are performed between those two species, the temperature

of the MC acceptance probability being set to the temperature of the underlying molecular dynamics run. A sample LAMMPS script for running such a simulation is provided in the supporting zenodo directory (see above for the address to the directory).

Warnings and dangerous situations

We describe here a few situations and systems where one should not expect the presented ACE Fe-O potential to perform well.

First of all, one should avoid running simulation of systems containing both Fe_{NM} and non-zero magnetic Fe atoms (*i.e.* $Fe_{\uparrow}$ and $Fe_{\downarrow}$). Indeed, since training the potential on such configurations without carrying constrained magnetism DFT calculations is really difficult, interactions between these magnetic Fe species are expected to be ill-defined. Usually the magnetic moments of all Fe atoms contained in the system tend to stabilize to either zero or non-zero magnetic moments. Coexistence of Fe_{NM} and Fe atoms with a non-zero magnetic moment can however occur when two Fe atoms are very close to each other.

Also the $Fe_{NM} - O$ interactions are not well described since most of the training data and stable configurations, relax to non-zero magnetic moments on the Fe atoms. Non-magnetic oxides are highly unstable, unless they are compressed at high pressures, where the $Fe_{NM} - O$ interactions are not expected to be ill-parameterized. We however did not test the potential in the pressure range where most iron oxides are non-magnetic.

Below distances of 1 Å, all interactions of the potential smoothly switch from ACE to a repulsive potential, which helps atoms not to fuse when coming too close to each other.

Supplementary references

[1] Z. S. Basinski, W. Hume-Rothery, and A. L. Sutton, Proc. R. Soc. London **229**, 459 (1955).

[2] M. Rinaldi, M. Mrovec, A. Bochkarev, Y. Lysogorskiy, and R. Drautz, npj Comput. Mater. **10**, 1 (2024).

[3] J. J. Adams, D. S. Agosta, R. G. Leisure, and H. Ledbetter, J. Applied Phys. **100**, 113530 (2006).

[4] L. De Schepper, D. Segers, L. Dorikens-Vanpraet, M. Dorikens, G. Knuyt, L. M. Stals, and P. Moser, Phys. Rev. B **27**, 5257 (1983).

[5] H. J. Wollenberger, Physical Metall. **2** (1996).

[6] P. I. Dorogokupets, A. M. Dymshits, K. D. Litasov, and T. S. Sokolova, Sci. Rep. **7**, 41863 (2017).

[7] R. Nazarov, T. Hickel, and J. Neugebauer, Physical Review B **82**, 224104 (2010).

[8] Y. Lysogorskiy, C. v. d. Oord, A. Bochkarev, S. Menon, M. Rinaldi, T. Hammerschmidt, M. Mrovec, A. Thompson, G. Csányi, C. Ortner, and R. Drautz, npj Comput. Mater. **7**, 1 (2021).

[9] L. Ventelon and F. Willaime, Philos. Mag. **90**, 1063 (2010).

[10] B. Bienvenu, L. Dezerald, D. Rodney, and E. Clouet, Acta Mater. **236**, 118098 (2022).

[11] I. Bleskov, T. Hickel, J. Neugebauer, and A. Ruban, Phys. Rev. B **93**, 214115 (2016).

[12] B. Sadigh, P. Erhart, A. Stukowski, A. Caro, E. Martinez, and L. Zepeda-Ruiz, Phys. Rev. B **85**, 184203 (2012).
